# A Double‐Armed, Hydrophilic Transition Metal Complex as a Paramagnetic NMR Probe

**DOI:** 10.1002/anie.201906049

**Published:** 2019-08-13

**Authors:** Qing Miao, Wei‐Min Liu, Thomas Kock, Anneloes Blok, Monika Timmer, Mark Overhand, Marcellus Ubbink

**Affiliations:** ^1^ Gorlaeus Laboratories Leiden Institute of Chemistry Leiden University Einsteinweg 55 2333 CC Leiden The Netherlands; ^2^ Department of Chemistry Fu Jen Catholic University No. 510, Zhongzheng Rd., Xinzhuang Dist., New Taipei City 24205 Taiwan

**Keywords:** magnetic susceptibility tensors, NMR spectroscopy, paramagnetic relaxation enhancements, pseudocontact shifts, transition metals

## Abstract

Synthetic metal complexes can be used as paramagnetic probes for the study of proteins and protein complexes. Herein, two transition metal NMR probes (TraNPs) are reported. TraNPs are attached through two arms to a protein to generate a pseudocontact shift (PCS) using cobalt(II), or paramagnetic relaxation enhancement (PRE) with manganese(II). The PCS analysis of TraNPs attached to three different proteins shows that the size of the anisotropic component of the magnetic susceptibility depends on the probe surroundings at the surface of the protein, contrary to what is observed for lanthanoid‐based probes. The observed PCS are relatively small, making cobalt‐based probes suitable for localized studies, such as of an active site. The obtained PREs are stronger than those obtained with nitroxide spin labels and the possibility to generate both PCS and PRE offers advantages. The properties of TraNPs in comparison with other cobalt‐based probes are discussed.

## Introduction

Ever since the determination of first metalloprotein structures using paramagnetic NMR restraints,^1^ it has been acknowledged that paramagnetism is a powerful tool for the study of biomolecules. The interactions of unpaired electrons with nuclei generate paramagnetic effects that contain structural information. The pseudocontact shift (PCS) and paramagnetic relaxation enhancement (PRE) are the paramagnetic effects that most frequently find application.^2^ PCS offer long‐range distance and conformational information and can be measured easily with high precision. PREs only yield distance information but are strongly distance‐dependent, making them exquisitely sensitive to minor states in which the nuclear‐electron distance is reduced.^3^ In proteins that bind metals naturally, a paramagnetic center is already present or can be introduced by replacing a diamagnetic metal ion, like Ca^II^ or Mg^II^, with a paramagnetic ion, such as Co^II^, Mn^II^, or a lanthanoid, Ln^III^.^4^ For other proteins, the introduction of a paramagnetic center is required, either through genetic means^5^ or chemical attachment. To limit the effects on the protein and obtain a single set of paramagnetic effects, the ideal chemical probe has no net charge and is hydrophilic, positioned rigidly relative to the protein, and of high symmetry.

Over the past years, many paramagnetic NMR probes were designed and synthesized.^6^ Ln^III^ has been the paramagnetic center of choice in many cases because of the range of anisotropic components of the magnetic susceptibilities (described by the Δχ tensor) that these ions display, with sizes of 2×10^−32^ m^3^ for Eu^III^, 8×10^−32^ m^3^ for Yb^III^ and up to 50–84×10^−32^ m^3^ for Tm^III^, Tb^III^, and Dy^III^.^7^ The similarity in coordination chemistry makes it possible to use the same probe with different Ln^III^ ions. Fewer probes for transition metals have been reported. Of the transition metal ions, high‐spin Co^II^ yields among the largest PCSs, with tensor sizes in the order of 2–7×10^−32^ m^3^,1c and displays weak PREs, whereas Mn^II^ causes strong PREs owing to the presence of five unpaired electrons and a long electronic relaxation time.^8^

The use of transition metal ions as paramagnetic centers for protein structural studies has already a long history.^9^ However, only few site‐specific transition metal probes have been designed for protein NMR studies. S‐(2‐pyridylthio)‐cysteaminyl‐EDTA is a commercially available (TRC, Toronto, Canada) transition metal probe. However, it yields multiple PCS for a nucleus owing to the presence of stereoisomers of the complex.^10^ Some other probes are attached to the protein through a single arm and require additional coordination by a residue of the protein near the attachment site.^11^ Recently, two‐point attachment was introduced for Co^II^ probes to ensure that the metal ion is rigid relative to the protein.^12^ For one of these probes, metal ion exchange between solvent and probe was observed.12a This prompted us to develop a double‐armed transition metal ion probe that could tightly bind the metal ion and generate a single set of paramagnetic effects. Cyclen (1,4,7,10‐tetraazacyclododecane) is a widely used building block for metal ligand design owing to its high metal binding affinity. A large number of cyclen derivatives have been developed for metal ions for the application in biomedicine^13^ and magnetic resonance imaging (MRI),^14^ for which high thermodynamic stability and kinetic inertness are required. Among these reported cyclen‐based complexes, there are, however, few successful Co^II^ and Mn^II^ complexes, in particular for Mn^II^, which can easily be oxidized by air.^15^

Herein, we report the design and synthesis of several C_2_‐symmetric cyclen derivatives, which can tightly bind the transition metal ions Co^II^ and Mn^II^ and are stable in air and buffers. These transition metal NMR probes (TraNPs) were tested using three proteins. The TraNPs are linked to the proteins trough two disulfide bonds at a specific location on protein surface, yielding single sets of paramagnetic effects in NMR spectra. Interestingly, the sizes of the Δχ tensor of Co‐TraNP are in the range of 2–5×10^−32^ m^3^ for different proteins, indicating that the protein environment has an indirect effect on the Co^II^ coordination that influences the Δχ tensor. We discuss the TraNP properties and compare them with other reported Co^II^ tags.

## Results and Discussion

**Figure 1:**
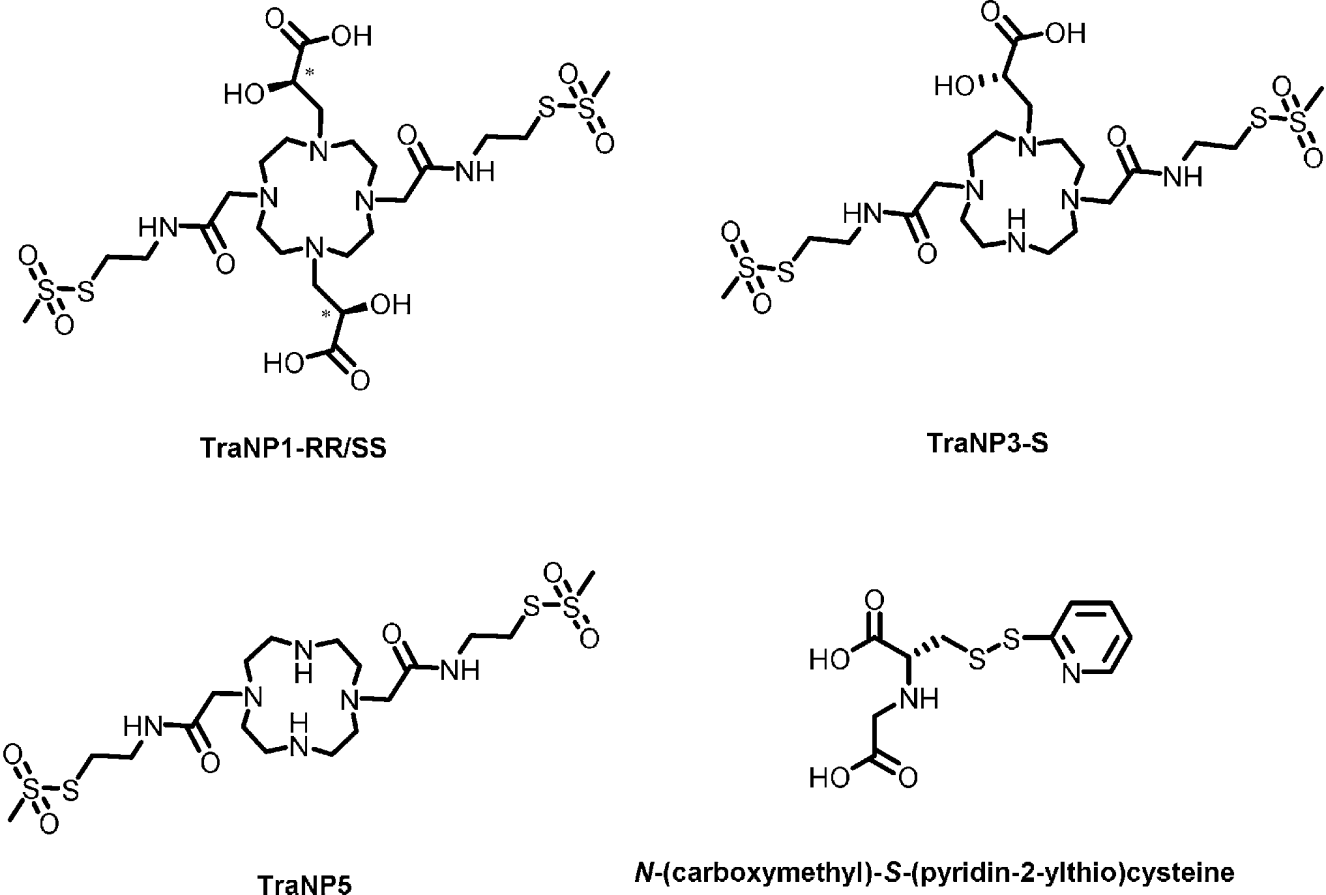
Chemical structures of TraNP1‐RR/SS, TraNP3‐S, TraNP5, and *N*‐(carboxymethyl)‐S‐(pyridin‐2‐ylthio) cysteine (tag 1). The asterisks in the structures of TraNP1‐SS/RR indicate the asymmetric carbons.

Derivatives of 1,4,7,10‐tetraazacyclododecane (cyclen) have been used extensively for metal binding because of their favorable metal coordination properties.^16^ For a two‐armed transition metal probe, C_2_‐symmetric derivatives that allow attachment to two cysteine residues are required.7b A selective partial protection and alkylation strategy was used to synthesize the novel C_2_‐symmetric metal binding ligands TraNP1‐SS and TraNP1‐RR, as well as the control compounds TraNP3‐S and TraNP5 (Figure 1 and the Supporting Information, Schemes S1 and S2). Except for TraNP5, which has poor affinity for metals ions, the metal complexes are stable under atmospheric condition. In addition, *N*‐(carboxymethyl)‐S‐(pyridin‐2‐ylthio)cysteine was synthesized (Figure 1, *tag 1*) and was used for comparison with TraNPs.12a

**Figure 2:**
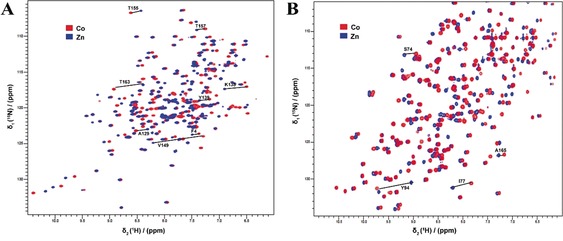
Overlay of ^1^H–^15^N HSQC spectra of Co^II^ (red) and Zn^II^ (blue) loaded TraNP1‐RR attached to A) T4Lys K147C/T151C and B) BCX E78Q/T109C/T111C. Several PCS are indicated with solid lines and residue numbers. The NMR spectra were recorded at 14.1 T (600 MHz).

Three proteins were used to characterize the paramagnetic properties of the TraNPs, T4 lysozyme (T4Lys) with the mutations K147C/T151C, *Bacillus circulans* xylanase (BCX) with mutations E78Q/T109C/T111C and ubiquitin with the mutations E24C/A28C. The mutation E78Q in BCX abolishes catalytic activity but is otherwise irrelevant for this study. These three proteins contain no cysteine residues apart from the pair introduced for probe attachment. In T4Lys and ubiquitin, the two cysteine residues are located in an α‐helix, whereas in BCX, they are in a β‐strand. The PCS caused by Co^II^ on the amide groups in the proteins were obtained by taking the difference of the ^1^H^N^ chemical shifts in the spectra of the Co^II^ and Zn^II^‐tagged proteins (Figure 2 and Figures S1 and S2). The PCS were fitted to Equation (S1) in the Experimental Section (Supporting Information) to obtain the two components of the anisotropic part of the magnetic susceptibility, Δχ_ax_ and Δχ_rh_, the orientation of the Δχ tensor and the position of the paramagnetic center relative to the protein structures.

For TraNP1 T4Lys (K147C/T151C), BCX (E78Q/T109C/T111C) and ubiquitin (E24C/A28C) more than 80, 100, and 40 PCS were measured and fitted against published structures. The results are presented in Table 1 and Tables S1–S3. A good correlation was found between the experimental and back‐calculated PCS (Figure S3). Only the PCS of the amide group of T4Lys residue 163 deviates strongly from the calculated value. This residue is located at the C‐terminus and its location in the structure may be ill‐defined. It was excluded from the calculations. The results for different structures of the same protein were essentially the same (Tables S1–S3).

**Table 1:** PCS‐based Δχ tensor parameters of Co^II^‐TraNP1 attached to T4Lys K147C/T151C, BCX E78Q/T109C/ T111C, and ubiquitin E24C/A28.

Protein probes	T4Lys		BCX		Ubiquitin
	**RR**	**SS**	**RR**	**SS**	**SS**
Δχ_ax_^[a]^	5.2±0.1	4.6±0.1	3.8±0.2	2.6±0.1	2.0±0.1
Δχ_rh_^[a]^	1.2±0.1	0.9±0.1	0.5±0.1	0.6±0.1	0.4±0.1
Restraints	81	89	105	100	45
Q_a_^[b]^	0.04	0.04	0.05	0.06	0.04
Co^II^–Cys Cα [Å]	7.6 (C147) 8.4 (C151)	7.9 (C147) 8.3 (C151)	9.8 (C109) 7.9 (C111)	9.8 (C109) 8.0 (C111)	8.9 (C24) 9.3 (C28)
PDB entry	2lzm^18^	2lzm^18^	2bvv^19^	2bvv^19^	2mjb^20^

[a] in 10^−32^ m^3^; [b] Adjusted Q‐value, see Eq. (S2).

**Figure 3:**
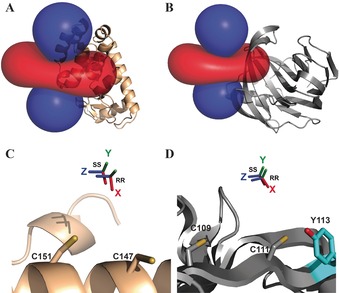
Δχ tensors of TraNP1. A,B) The ±0.2 ppm PCS iso‐surfaces of TraNP1‐RR are plotted for T4Lys K147C/T151C (PDB entry 2lzm; A) and BCX E78Q/ T109C/T111C (PDB entry 2bvv; B). The backbones of the proteins are drawn in ribbon representation. Positive and negative PCS are indicated by blue and red iso‐surfaces, respectively. C,D) Co^II^ positions and tensor orientations of TraNP1‐RR and TraNP1‐SS are shown for T4Lys K147C/T151C (C) and BCX E78Q/T109C/T111C (D). The Cys residues used for attachment have been modelled in the structure and are shown as sticks. The sidechain of Tyr113 in BCX is shown as sticks.

The iso‐surfaces of the Δχ tensors, plotted on T4Lys, BCX, and ubiquitin, are shown in Figure 3 and Figure S4. The Δχ tensors are mostly axial, with only a minor rhombic component. The iso‐surfaces of the two enantiomers are very similar. The Co^II^ ions are located between the cysteine residues, as expected for a two‐armed probe, and in line with results obtained for the lanthanoid probes, CLaNP5 and CLaNP7.7b, 17 The distances between the Co^II^ and the cysteine C_α_ atoms are between 7.6 and 9.8 Å (Table 1). The distance between Co^II^ ions in TraNP1‐RR and TraNP1‐SS bound to T4Lys is only 1.4 Å and the Δχ tensor orientations are similar (Figure 3). However, the Δχ_ax_ of TraNP1‐SS is 12 % smaller than that of TraNP1‐RR (Table 1). Also on BCX, TraNP1‐RR and TraNP1‐SS position the Co^II^ ions in similar locations and the Δχ tensor frame orient in the same way. In this case, Δχ_ax_ of TraNP1‐SS is even 32 % smaller than for TraNP1‐RR. Interestingly, the Δχ_ax_ of TraNP1‐RR (SS) attached to BCX is 27 % (44 %) smaller than for this probe attached to T4Lys (Table 1). For ubiquitin, the Δχ tensor of Co‐TraNP1‐SS labeled on ubiquitin was even smaller than for the other two proteins (Table 1). These differences will be discussed later.

**Figure 4:**
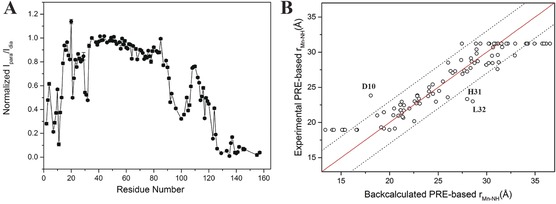
PRE. A) I_para_/I_dia_ ratios of amide resonances in ^1^H–^15^N HSQC spectra of T4Lys K147C/T151C tagged with Mn^II^/Zn^II^‐TraNP1‐SS. B) PRE‐derived Mn^II^–^1^H^N^ distances plotted vs. the Co^II^–^1^H^N^ distances with the Co^II^ position derived from PCS data. No fitting was performed. The solid red line represents a perfect correlation and the dotted lines show ±3 Å margin.

To investigate whether TraNP1 can also be used to generate PREs with a transition metal, TraNP1‐SS was loaded with Mn^II^ and linked to T4Lys K147C/T151C. PREs were obtained by comparing the intensities of amide resonances in HSQC spectra of Mn^II^ and Zn^II^‐tagged T4Lys samples (Figure S5). Figure 4 A shows the intensity ratios. From these, the PREs and Mn^II^–^1^H^N^ distances were derived [Supporting Information, Experimental Section, Eqs. (2) and (3)]. These distances were compared with those obtained from the PCS‐based Co^II^ position, assuming that Mn^II^ and Co^II^ in TraNP1‐SS sit in the same position relative to T4Lys. A good correlation, with a margin of ±3 Å, for distances between 19 and 31 Å was found (Figure 4 B). For peaks that broadened beyond detection, the observed distance was set to 19 Å and for the amide groups with unaffected peak intensities the distance was set to 31 Å, explaining the points on these two horizontal distance lines in Figure 4 B. Three clear outliers were observed (residues 10, 31, and 32). It is not obvious why these amide groups give deviating results. The assignments appear correct, and the structure is well‐defined for these residues. The position of the Mn^II^ ion was also determined by fitting it to the experimental distances (see Supporting Information for details). These calculations place the Mn^II^ ion 2.5 Å away from the Co^II^ position based on the PCS data (Figure S6 A). The experimental and back‐calculated distances correlate within the ±3 Å range for most residues, except for residues 10, 31, and 32, which deviate 4–5 Å (Figure S9 B). Exclusion of these three residues yielded a better fit (Figure S6 C). These and other calculations showed that the exact calculated position of the Mn^II^ ion is strongly dependent on the input data set (Figure S6 A). It is estimated that the PRE‐based position of the metal has a precision of 2–3 Å and is less precise than the location based on the PCS data. However, the metal positions obtained through both approaches are consistent, being in between the two cysteine residues.

To determine which of the pending arms coordinate the metal in TraNP1, TraNP3‐S and TraNP5 were synthesized, lacking one or both hydroxy‐propionic acid groups, respectively. TraNP5 failed to bind metals, whereas TraNP3‐S was capable of coordinating Co^II^. When this complex was linked to T4Lys, two sets of PCS were observed for each amide group and the PCS were smaller than those observed for TraNP1 (Figure S7 A). As compared to TraNP1, the C_2_‐symmetry is broken in TraNP3, resulting in two isomers upon attachment to the protein, which could be the cause of the double resonances. It suggests that the amide groups in the other two pending arms are incapable of coordinating the Co^II^ ion, so these arms have additional rotational freedom compared to the coordination arms of the lanthanoids binding counterparts, CLaNP5 and CLaNP7.7b, 17 Lanthanoids require eight or nine ligands, so all pending arms are involved in metal coordination. We also tested lanthanoid binding to TraNP1. The affinity for these metals is poor.

To compare TraNP1 with another two armed Co^II^‐tag, we synthesized the published Co^II^ probe named tagging agent 1 (tag 1), *N*‐(carboxymethyl)‐S‐(pyridin‐2‐ylthio)cysteine, Figure 1. In this case, the two cysteine residues on the protein each react with one probe molecule and the Co^II^ ion is sandwiched in between the two attached groups. Swarbrick et al.12a attached this probe to ubiquitin E24C/A28C and reported a remarkably large Δχ tensor (−7.4×10^−32^ m^3^). We repeated the experiment with the same ubiquitin variant and also attached tag 1 to T4Lys K147C/T151C. Labeling was confirmed with mass spectrometry (Figures S8 and S9). For all the samples linked to either tag 1, Co^II^‐tag 1, or Zn^II^‐tag 1, the same mass was observed, of the free protein plus 409 Da. This mass difference equals the mass of two attached tag 1 molecules (354 Da) plus an additional 55 Da. As this extra mass was present independent of the presence of either Co^II^ or Zn^II^ in the sample, we assume that tag 1 loses these metal ions and picks up additional mass in the process of the mass spectroscopy measurement, for example, Fe^III^ or Mn^II^ ions. ^1^H‐^15^N HSQC spectra of the Co^II^ and Zn^II^‐tagged ubiquitin and T4Lys were recorded and the PCS measured. The size and orientation of the Δχ tensor of tag 1 derived from the experimental PCS analysis of the ubiquitin NMR spectra were the same as those obtained using the published PCS values (Figures S10 A and S11 and Table S4).

In our spectra, some of the residues showed more than a single paramagnetic peak, such as residues K6, T7, and H68 (Figure S11). Moreover, Co^II^ loading appeared to be incomplete. In the NMR spectra of ubiquitin tagged with Co^II^‐loaded tag 1, diamagnetic peaks were present (Figure S11 B), even if 1.2 equiv of Co^II^ was added, rather than the reported 0.6 equiv. The tagged, metal‐free ubiquitin also behaved curiously, showing many double peaks in the HSQC spectrum, compared to untagged ubiquitin. Upon addition of Zn^II^, single peaks remain in the HSQC spectrum (Figure S11 C). Thus, the metal‐free tag 1 caused the presence of two forms of the protein. Also for T4Lys K147C/T151C, tag 1 showed partial loading with Co^II^ or Zn^II^, even with 10 equiv of the metal ion added. Again, more than one peak with PCS were observed for some of the residues (Figure S12). From the tag 1‐T4Lys HSQC spectrum, around 50 PCS were measured and used for Co^II^ positioning and Δχ tensor calculation. The Co^II^ is located between the two cysteine residues and the distances to the two cysteine C_α_ atoms are around 7 Å (Figure S10 B), which is similar to the results for the ubiquitin variant. As observed with TraNP1, the size of the tensor was affected by the protein because the Δχ_ax_ component of Co^II^ *‐*tag 1 was, though still quite large, somewhat smaller than for the ubiquitin variant, and the Δχ_rh_ component was more than three times smaller (Table S4).

The synthesis and characterization of a new two‐armed transition metal binding NMR probe, TraNP1, are reported. Loaded with Co^II^ and attached to the proteins T4Lys, BCX and ubiquitin through two disulfide bridges, it causes PCS of the resonances of amide nuclei. The PCS can be fitted well and yield the position of the metal relative to the protein as well as the orientation and size of the Δχ tensor. Whereas metal position and tensor orientation are similar, interesting differences are observed in the sizes of the Δχ_ax_ and Δχ_rh_ between the two enantiomers, as well as between T4Lys, BCX, and ubiquitin. We are puzzled by the large variation in these tensor sizes for cobalt ions that are expected to have the same coordination environments. We attribute these effects to slight differences in coordination of the cobalt ion. The binding of the probe to the protein and interactions with protein side‐chains may introduce slight strain on the Co^II^ ligands, leading to changes in the electron distribution and thus in the paramagnetic effect. It is likely that the cobalt ion is coordinated by the four ring nitrogen atoms and two carboxy oxygen atoms in a pseudo‐octahedral conformation.

**Figure 5:**
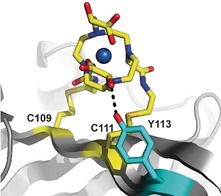
Model of Co‐TraNP1‐SS attached to BCX E78Q/T109C/T111C. The protein is represented in cartoon mode and the two cysteine residues and the tag were modelled in the structure (PDB entry 2bvv).^19^ The oxygen of Tyr113 (in cyan sticks) can readily be brought into hydrogen bond distance of one of the hydroxy groups of TraNP1‐SS. The cysteine residues and the probe are shown in sticks, with carbon atoms in yellow and the nitrogen, oxygen, and sulfur atoms in blue, red, and dark yellow, respectively. The Co^II^ ion is shown as a sphere.

Based on the crystal structure of a similar compound,^21^ the structure of TraNP1 was modelled using Spartan′14 & Spartan′14 Parallel Suite (www.wavefun.com). The two enantiomers indeed show slight differences (Figure S13). The linkage of TraNP1 to the proteins was also modelled, using XPLOR‐NIH.^22^ In this model, the coordination obtained in the Spartan model was constrained and the arms for attachment were free to rotate. The protein backbone was fixed, and side chains were allowed to rotate. The position of the Co^II^ ion was restrained to the experimental position. An acceptable model was obtained in which the plane of the cyclen ring is roughly perpendicular to the surface of the protein and the arms for attachment point away from the ring, relative to the hydroxy‐propionic acid groups (Figure 5). In the BCX‐TraNP1‐SS model, Tyr113 is located within hydrogen‐bonding distance of one of the hydroxy groups. We speculate that such an interaction with a side chain could affect the ligand coordination of the cobalt ion and influence the size of the Δχ tensor. It is concluded that the paramagnetic properties of Co^II^ are very sensitive to the environment of the ligands, strongly affecting the size of the anisotropic component of the magnetic susceptibility, in line with the large differences reported for the size of paramagnetic effects in other Co^II^‐compounds.4a, 11a, 23

**Figure 6:**
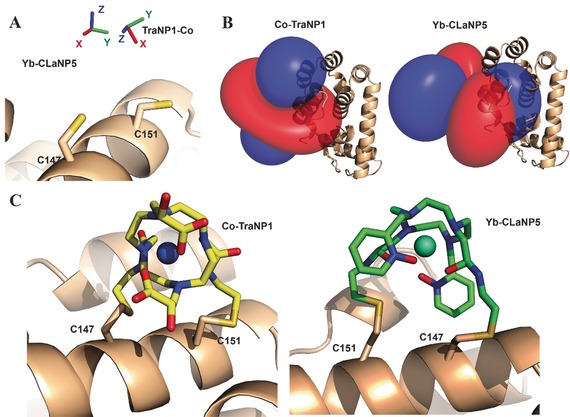
Comparison of Co^II^‐TraNP1‐SS and Yb^III^‐CLaNP5 attached to T4Lys K147C/T151C. A) Metal positions and tensor rotations, B) PCS iso‐surfaces (±0.4 ppm for Yb^III^‐CLaNP5, ±0.2 ppm for Co^II^‐TraNP1‐SS), and C) models of the protein‐probe structures. The protein is shown in ribbon representation, the probes and cysteines are shown in sticks. The metals are shown as spheres.

These observations are strikingly different from lanthanoid probes. For rigidly attached probes, such as CLaNP‐5 and CLaNP‐7, usually similar sizes for Δχ_ax_ and Δχ_rh_ are found, independent of site of attachment.7b, 17 As a consequence, the Δχ_ax_ and Δχ_rh_ need to be determined for TraNP1 in each system, whereas for CLaNP, the sizes can, to first approximation, be estimated on the basis of literature values. Figure 6 presents a comparison of the results for Co^II^‐TraNP1‐SS and Yb^III^‐CLaNP5 attached to T4Lys at residues 147 and 151.^24^ The metals are 2.6 Å apart, though both are located between the Cys residues. Also, the direction of the *z*‐axis of the tensor and the degree of rhombicity differ considerably. Figure 6 C shows the models of the probes attached to T4Lys. In CLaNP, all the four pending arms are involved in the coordination of the metal and thus are oriented in the same direction, placing them in such a way that the cyclen ring is roughly parallel to the protein surface and the metal in between the cyclen ring and the protein. In TraNP, the cyclen ring is perpendicular to the surface and the metal is thus on one side of the ring, relative to the protein.

The comparison of TraNP1 and tag 1 confirmed that the coordination of the Co^II^ atom has a large effect on the size of the Δχ tensor. For tag 1, four carboxyl groups and two secondary amines are likely involved in the coordination, whereas TraNP1 has two carboxyl groups and four tertiary amines contributing to the coordination. However, both tags are likely to provide a distorted octahedral environment, so it is unclear whether the different type of ligands are the cause of the large Δχ tensor of tag 1, yielding larger PCS than obtained with TraNP1 and approaching those obtained with Yb^III^ probes. The metal is roughly located at the same position in both probes, in between the cysteines and 7–8 Å away from the C_α_ atoms. However, the tensor axes are oriented very differently (Figure S10).

A disadvantage of tag 1 is that some amides show more than one resonance in the spectrum of the paramagnetic sample. Furthermore, the metal affinity appears to be relatively low, leading to the presence of peaks of metal‐free tagged protein in the spectra of the paramagnetic sample, increasing spectral crowding that could be problematic for larger proteins. Next to the doubly anchored tag 1, several tags with a single attachment point were reported, like (2‐pyridylthio)‐cysteamine‐EDTA, 2‐vinyl‐8‐hydroxyquinoline (2V‐8HQ),11a and 3‐mercapto‐2,6‐pyridinedicarboxylic acid (3MDPA).11b As a commercially available probe, (2‐pyridylthio)‐cysteamine‐EDTA is widely used in protein paramagnetic NMR after it was first reported with Fe^III^ as the paramagnetic center.^25^ Further research found that the Co^II^‐loaded (2‐pyridylthio)‐cysteamine‐EDTA generated two sets of PCS, in one case^10^ but not in other,^26^ owing to the presence of stereoisomers of the complex. The 2V‐8HQ is a rigid and small probe for Co^II^ and requires additional ligands from a protein side‐chain, making the metal location less predictable than for a two‐armed probe. Slow exchange of Co^II^ ions between the solvent and the 2V‐8HQ tag on ubiquitin was reported. The affinity and exchange rate depend on the coordinating side chain. For 3MDPA, which can bind Ln^III^ and Co^II^, the tensor orientations for all the metal ions are similar but the metal affinity is very weak.

To reduce the probe attachment flexibility, genetic incorporation of natural or unnatural amino acids in the protein sequence has been proposed. The unnatural amino acid bipyridylalanine (BpyAla)^27^ and 2‐amino‐3‐(8‐hydroxyquinolin‐3‐yl)propanoic acid dihydrochloride (HQA)^28^, which both have a side chain strongly chelating transition metal ions, were successfully introduced into West Nile virus NS2B‐NS3 protease (WNVpro) and membrane proteins (1TM‐CXCR1 and p7), respectively. Similar to 2V‐8HQ, both require additional ligands provided by protein side chains. HQA was used for Mn^II^ to measure PRE. Recently, it was reported that also a dihistidine (diHis) motif can be used to bind Co^II^.12b The motif was introduced to ubiquitin on an α‐helix, as well as a β‐strand of GB1. Also in this study, the dHis motif generated different Δχ tensor values and orientations for the different protein variants.

## Conclusions

TraNP1 adds a new probe to the range of paramagnetic probes available for NMR on biomolecules.2e, 6a Co^II^ has a smaller anisotropic magnetic susceptibility than most lanthanoids, placing it close to Pr^III^.4b Its application can lie in studying small and local structural changes in proteins and protein complexes, such as can occur in enzyme active sites during catalysis or in protein pockets upon ligand binding. The effects of stronger lanthanoids are less suitable for studying such small structural changes. The reason is that such probes cannot detect changes close to the probe owing to PRE effects and events further away require larger structural changes to cause significant PCS changes. A 1 Å change at a large distance from a paramagnetic metal leads to a small relative change in angle and distance and thus a small change in PCS, both in absolute and relative terms, even for strongly paramagnetic ions. This point is illustrated with an example in Table S5. Thus, Yb^III^ and the stronger Ho^III^, Dy^III^, Tm^III^, and Tb^III^ are suited for studying domain motions and determination of structures of complexes,2e whereas probes with smaller Δχ tensors are suitable for detection of nearby, small structural changes. The relatively low paramagnetic anisotropy of Co‐probes makes the measurement of paramagnetically induced RDC inconvenient at routine fields, such as 14 T (600 MHz), with maximal predicted values for ^1^H‐^15^N of 1.7 and 4 Hz for TraNP1 and tag 1, respectively. At the highest fields achievable, obtaining these RDC becomes realistic (7 and 16 Hz, respectively, at 28 T, 1200 MHz), offering possibilities for the study of protein mobility. Finally, the fact that TraNP1 can also bind Mn^II^ to measure PRE is convenient because PRE‐derived distances complement the restraints obtained from PCS.

## Conflict of interest

The authors declare no conflict of interest.

## Supporting information

As a service to our authors and readers, this journal provides supporting information supplied by the authors. Such materials are peer reviewed and may be re‐organized for online delivery, but are not copy‐edited or typeset. Technical support issues arising from supporting information (other than missing files) should be addressed to the authors.

Supplementary
