# Supplementary material for: A Double‐Armed, Hydrophilic Transition Metal Complex as a Paramagnetic NMR Probe
Source: Angew Chem Int Ed Engl. 2019 Aug 13;58(37):13093–100. doi: 10.1002/anie.201906049 (PMC6771572; doi:10.1002/anie.201906049)

Angewandte Chemie
Eine Zeitschrift der Gesellschaft Deutscher Chemiker

Supporting Information

A Double-Armed, Hydrophilic Transition Metal Complex as a Paramagnetic NMR Probe

*Qing Miao, Wei-Min Liu, Thomas Kock, Anneloes Blok, Monika Timmer, Mark Overhand, and Marcellus Ubbink**

anie_201906049_sm_miscellaneous_information.pdf

Supporting information

Contents

Synthesis Section S3

Protein labeling S9

Protein NMR spectroscopy S9

PCS and PRE calculation S10

Scheme S1. Synthesis of TraNP1 .... S12

Scheme S2. Synthesis of TraNP3-S and TraNP5 .... S12

Table S1. PCS-based Δχ-tensor parameters of Co(II)-TraNP1 attached to T4Lys K147C/T151C, fitted to three crystal structures. S13

Table S2. PCS-based Δχ-tensor parameters of Co(II)-TraNP1 attached to T109C/T111C BCX, fitted to three crystal structures S14

Table S3. PCS-based Δχ-tensor parameters of Co(II)-TraNP1-SS attached to E24C/A28C ubiquitin, fitted to three crystal structures. S15

Table S4. PCS-based Δχ tensor parameters of *tag 1* and TraNP1-SS attached to ubiquitin E24C/A28C and T4Lys K147C/T151C S16

Table S5. Predicted PCS changes for some paramagnetic metals upon small changes in the position of the nucleus relative to the Δχ tensor frame S17

Figure S1. Overlay of ^{1}H-^{15}N HSQC spectra of Co(II) and Zn(II) loaded TraNP1-SS attached to T4Lys K147C/T151C S18

Figure S2. Overlay of ^{1}H-^{15}N heteronuclear correlation spectra of Co(II) and Zn(II) loaded TraNP1-SS attached to ubiquitin E24C/A28C S19

Figure S3. Experimental $^{1}H^{N}$ PCS (ppm) of Co(II)-TraNP1 were plotted against the back-calculated values S20

Figure S4. PCS iso-surfaces of TraNP1-SS plotted on the structures of T4Lys K147C/T151C S21

Figure S5. Overlay of ^{1}H-^{15}N HSQC spectra of Mn(II)-TraNP1-SS and Zn(II)-TraNP1-SS attached to T4Lys K147C/T151C S22

Figure S6. PRE data analysis S23

Figure S7. Overlay of ^{1}H-^{15}N HSQC spectra for various TraNPs attached to T4Lys K147C/T151C. S24

Figure S8. ESI-TOF mass spectra of 15N-enriched E24C/A28C ubiquitin mutant linked to *tag 1*, Co-*tag 1*and Zn-*tag 1* S25

Figure S9. ESI-TOF mass spectra of 15N-enriched T147C/K151C T4Lys mutant linked to *tag 1*, Co-*tag 1* and Zn-*tag 1* S26

Figure S10. Metal positions and tensor orientations of Co(II)-TraNP1-SS and Co(II)-tag 1 attached to ubiquitin E24C/A28C (A) and T4Lys K147C/T151C S27

Figure S11. Overlay of ^{1}H-^{15}N HSQC spectra of Co(II)-*tag 1*, Zn(II)- *tag 1*, metal-free *tag 1* linked to ubiquitin E24C/A28C and untagged ubiquitin E24C/A28C S28

Figure S12. Overlay of ^{1}H-^{15}N HSQC spectra of T4Lys K147C/T151C linked to Co(II) or Zn(II) loaded *tag 1*. S29

Figure S13. Structure models of TraNP1 .... S30

References S31

Appendixes S33

Appendix S1 ESI-TOF mass spectra of free ^{15}N-enriched K147C/T151C T4Lys mutant and linked to Co-TraNP1 .... S33

Appendix S2. ESI-TOF mass spectra of 15N-enriched free T109C/T111C BCX mutant and linked to Co(II)-TraNP1 .... S34

Appendix S3. ESI-TOF mass spectra of 15N-enriched E24C/A28C ubiquitin mutant and linked to Co-TraNP1 .... S35

Appendixes S4 $^{1}H^{N}$ PCS of Co-TraNP1-SS linked to BCX S36

Appendixes S5 $^{1}H^{N}$ PCS of Co-TraNP1-RR linked to BCX S38

Appendixes S6. $^{1}H^{N}$ PCS of Co-TraNP1-SS linked to T4Lys S40

Appendixes S7 $^{1}H^{N}$ PCS of Co-TraNP1-RR linked to T4Lys S42

Appendixes S8 $^{1}H^{N}$ PCS of Co-TraNP1-SS linked to ubiquitin S44

Appendixes S9 $^{1}H^{N}$ PCS of Co-*tag 1* linked to ubiquitin S45

Appendixes S10. $^{1}H^{N}$ PCS of Co-tag 1 linked to T4Lys S46

Appendixes S11 XPLOR script for PRE calculation parameters S47

Appendixes S12 Chemical NMR spectra S47

Synthesis Section

General: cyclen, 2-(aminoethyl)methanethiosulfonate hydrobromide, methyl (S)-oxirane-2-carboxylate, N-(tert-Butoxycarbonyloxy)succinimide, $Ln(OAc)_3$, $CoCl_2{\cdot}6H_2O$, ZnI_2, $MnCl_2{\cdot}4H_2O$, methyl (R)-oxirane-2-carboxylate and all other chemicals were purchased and used without further purification. Solvents were purchased from Honeywell, BIOSOLVE or Aldrich and directly used for synthesis. Superdex 75 columns and Sephadex G-25 PD10 desalting columns were purchased from GE Healthcare. Reactions were followed by TLC analysis on silica gel (F 1500 LS 254 Schleicher and Schuell, Dassel, Germany) and visualized by UV and/or ninhydrin, $KMNO_4$. Flash chromatography was performed with Screening Devices silica gel 60 (0.04-0.063 mm). A Waters preparative RP-HPLC system, equipped with a Waters C18-Xbridge 5 µm OBD (30 x 150 mm) column and Äkta Basic FPLC (GE Healthcare Inc.) system were used for purification. NMR spectra were recorded on a Bruker AV-400 (400/100 MHz), AV-500 (500/125 MHz) or AV-600 (600/150 MHz) spectrometer. A LCQ liquid chromatography mass spectrometry system and a Finnigan LTQ Orbitrap system were used for high-resolution mass spectrometry and protein conjugation analysis. A Thermo Finnigan LCQ Advantage MAX was used for liquid chromatography (LC)/mass spectrometry (MS) analysis. A Thermo Scientific™ NanoDrop 2000 spectrophotometer was used for protein concentration measurement.

S-(2-(2-bromoacetamido)ethyl) methanesulfonothioate, compound 1

To a solution of S-(2-aminoethyl) methanesulfonothioate-HBr salt (0.5 g, 2.13 mmol) in H_2O (20 mL) K_2CO_3 (0.59g, 4.26 mmol) was added. While stirring was continued, the reaction mixture was cooled (0°C) and a solution of acetyl bromide (0.3 mL, 4.26 mmol) in DCM (20 mL) was added drop-wised. The resulting two layers were separated and the water layer was extracted with ethyl acetate (3 x 20 mL). The organic layer (combination of the DCM layer and extracted ethyl acetate layer) was dried (Na_2SO_4), concentrated under reduced pressure and purified by silica flash column

chromatography, giving compound **1** as a white solid (1 g, 72% yield). R_f = 0.4 (pentane/ethyl acetate 4:1); mp: 53.2-53.6 ºC; ^{1}H NMR (400 MHz, $CDCl_3$, 293K): δ = 3.31-3.34 (t, 2H, $^2J_{H,H}$= 4 Hz, CH_2SSO_2), 3.36 (s, 3H, CH_3SSO_2), 3.62-3.67 (q, 2H, $^3J_{H,H}$ =8 Hz, CH_2NHCO), 3.86 (s, 2H, CH_2Br), 7.10 (b, 1H, NHCO); ^{13}C NMR (400 MHz, $CDCl_3$, 293K): δ = 28.89 (CH_3Br), 35.38 (CH_2SO_2), 37.33 (CH_2 CH_2SO_2), 39.87 (CH_2Br), 50.67 (CH_3SSO_2), 166.56 (CONH); HR-MS (ESI): m/z 297.9175 $[M+Na]^+$, calcd [$C_5H_{10}BrO_3S_2$] 297.9183; FTIR ν (cm^{-1}): 3291.2 (w), 1652.7 (s), 1539.7 (s), 1405.2 (w) , 1309.3 (s), 1209.2 (m), 1127.6 (vs), 957.3 (m), 745.5 (m).

Di-tert-butyl4,10-bis((*R*)-2-hydroxy-3-methoxy-3-oxopropyl)-1,4,7,10-tetraazacyclododecane-1,7-dicarboxylate, compound 3*RR*

To a solution of compound **2**[1] (1 g, 2.69 mmol) in methanol (30 mL) methyl (*R*)-oxirane-2-carboxylate (1.4 mL, 16.14 mmol) was added, and stirring was continued for 36 h at room temperature (r.t.), then the product was concentrated under reduced pressure and purified by silica flash column chromatography (40% of ethyl acetate in pentane) to give compound **3** **(*RR*)** as an amorphous white solid (1 g, 61% yield). An analogous procedure was used for synthesis of RR-isomer, 2 g (5.4 mmol) of compound **2** and 2.8 mL (16.14 mmol) of (*R*)-oxirane-2-carboxylate, with a yield of 63% (1.87 g) of compound **3** (***SS***). R_f = 0.35 (pentane/ethyl acetate 3:4); SS-isomer $[\alpha]_D^{20}$= -17.5 (C= 2 mg/mL, methanol), RR-isomer $[\alpha]_D^{20}$ = 16.5 (C = 10 mg/mL, methanol); RR-isomer ^{1}H NMR (500 MHz, $CDCl_3$, 298 K): δ = 1.44 (s, 18H, $(CH_3)_3C$), 2.68-2.67 (br, 6H, , CH_2N), 2.85 (br, 2H, CH_2CHOH), 2.99 (br, 4H, CH_2N), 3.32-3.33 (br, 8H, CH_2NH), 3.75 (s, 6H, CH_3OOC) 4.34 (br, 2H, $CHOHCH_2$); ^{13}C NMR (500 MHz, $CDCl_3$, 298K): δ = 28.57 ($(CH_3)_3C$), 48.73 (CH_2N), 52.47 (CH_3OOC), 55.10 (CH_2N), 58.69 (CH_2CHOH), 68.92 (CHOH), 80.26 (C $(CH_3)_3$)*,* 156.47 (COO*t*Bu); FTIR ν (cm^{-1}) RR-isomer: 2923.4 (w), 1684.2 9vs), 1734.4 (m), 1249 (w), 1159.1 (vs), 1054.6 (w), 1033.1 (w), 1013.1 (w); The spectroscopic data of the SS-isomer are in accordance with its enantiomer; RR-isomer HR-MS (ESI): m/z 577.3455 $[M+H]^+$, calcd [$C_{26}H_{48}N_4O_{10}$]

577.3404; SS-isomer HR-MS (ESI): m/z 577.3450 $[M+H]^+$, calcd $[C_{26}H_{48}N_4O_{10}]$ 577.3404.

Dimethyl3,3'-(4,10-bis(2-((2-((methylsulfonyl)thio)ethyl)amino)-2-oxoethyl)-1,4,7,10-tetraazacyclododecane-1,7-diyl)(2S,2'S)-bis(2-hydroxypropanoate), compound 4*RR*

Compound **3 (*RR*)** (0.5 g, 0.87 mmol) was dissolved in a mixture of dichloromethane and TFA (2.5 mL, 2:3 *v/v*) and stirred for 16 h at room temperature. The reaction mixture was concentrated under reduced pressure and co-evaporated with toluene to remove the TFA. The crude mixture was dissolved in acetonitrile (9 mL), and S-(2-(2-bromoacetamido)ethyl) methanesulfonothioate (0.61 g, 2.61 mmol), K_2CO_3 (0.48 g, 3.48 mmol) and a catalytic amount of tetrabutylammonium iodide (TBAI) were added. After stirring for 48 h at r.t., the reaction mixture was filtered and the solid was washed with acetonitrile (50 mL). The filtrate was concentrated under reduced pressure to give a yellow oil which was purified by reverse phase HPLC (0.2% TFA and a 10-20% acetonitrile gradient on a C18 preparative column) obtaining compound **4 (*RR*) (**0.42 g, 63%). A similar procedure was used for the synthesis of the SS-isomer, 1g (1.74 mmol) of compound **3** (***SS***) and 1.22 g (5.22 mmol), with a yield of 60% (0.8 g) of compound **4** (***SS***). RR-isomer $[\alpha]_D^{20}$= 16.3 (C= 10 mg/mL, Methnol); SS-isomer $[\alpha]_D^{20}$= -17.4 (C= 5 mg/mL, Methnol); RR-isomer ^1H NMR (500 MHz, CD_3OD, 333K): δ = 3.13 (br, 8H, CH_2N), 3.36-3.39 (t, 4H, $^2J_{H,H}$= 5 Hz, CH_2NHO), 3.43 (s, 8H), 3.47-3.55 (br, 8H, CH_2N), 3.52-3.61 (br, 4H, CH_2CHOH), 3.56-3.63 (m, 10H), 3.80 (s, 6H, CH_3COO), 4.77-4.81 (dd, 2H, $^1J_{H,H}$= 5 Hz, CHOH); ^{13}C NMR (500 MHz, CD_3OD, 333K): δ = 36.44 (CH_2SO_2), 40.37 (CH_2 CH_2SO_2), 50.65 (CH_2N), 52.51 (CH_2N), 53.35 (CH_3S), 55.94 (CH_2CONH), 56.97 (CH_2CHOH), 67.47 (CHOH), 172.74 ($COOCH_3$); FTIR (cm^{-1}) RR-isomer: 1748.6 (m), 1669.9 (s), 1313.6 (s), 1200.6 (m), 1177.7 (m), 1129.0 (s), 960.2 (w), 831.4 (w), 801.3 (w), 748.4 (w), 719.8 (w); The spectroscopic data of the SS-isomer are in agreement with its enantiomer; HR-MS (ESI) SS-isomer:

m/z 767.2468 $[M+H]^+$, calcd $[C_{26}H_{50}N_6O_{12}S_4]$ 767.2403; RR-isomer: m/z 767.2466 $[M+H]^+$, calcd $[C_{26}H_{50}N_6O_{12}S_4]$ 767.2403.

Di-tert-butyl4,10-bis(2-((2-((methylsulfonyl)thio)ethyl)amino)-2-oxoethyl)-1,4,7,10-tetraazacyclododecane-1,7-dicarboxylate, compound 5

To a solution of compound **2**[1a, 2](1 g, 2.69 mmol) in acetonitril (30 mL), compound **1** (3 g, 10.76 mmol) and K_2CO_3 (1.5 g, 10.76 mmol) were added. The reaction mixture was stirred at r.t. for 48 h, then filtered and concentrated under reduced pressure and purified by silica flash column chromatography (3% of MeOH in DCM) to give an oily product (1.1 g, 54% yield). R_f = 0.45 (DCM/MeOH 15:1); ^{1}H NMR (500 MHz, $CDCl_3$, 298K): δ = 1.45 (s, 18H, $(CH_3)_3C$), 2.84 (s, 8H, CH_2N), 3.23 (s, 4H, CH_2CONH), 3.34 (t, 4H, CH_2SO_2S), 3.37 (s, 6H, CH_3SSO_2), 3.37-3.38 (br, 8H, CH_2N), 3.60-3.64 (dd, 4H, $^2J_{H,H}$= 5 Hz, $CH_2CH_2SO_2S$); ^{13}C NMR (500 MHz, $CDCl_3$, 298K): δ = 28.71 $(CCH_3)_3C$), 35.86 (CH_2SO_2S), 39.03 ($CH_2CH_2SO_2S$), 80.59 ($C(CH_3)_3$), 156.70 (COO*t*Bu); FTIR (cm^{-1}): 2973.5 (w), 2933.4 (w), 1684.2 (vs), 1319.3 (s), 1156.2 (s), 1053.2 (w), 1033.1 (m), 960.2 (w), 831.2 (w), 799.2 (w), 748.4 (w), 721.2 (w); HR-MS (ESI): m/z 763.2850 $[M+H]^+$, calcd $[C_{28}H_{55}N_6O_{10}S_4]$ 763.2862.

(2S,2'S)-3,3'-(4,10-bis(2-((2-((methylsulfonyl)thio)ethyl)amino)-2-oxoethyl)-1,4,7,10-tetraazacyclododecane-1,7-diyl)bis(2-hydroxypropanoic acid), TraNP1

Compound **4** ***(RR)*** (0.2 g, 0.26 mmol) was dissolved in 1 M HCl (6 mL) and heated to 50°C for 8 h. The pH of the reaction mixture was adjusted to 6 by using a solution of sat. $NaHCO_3$ at 0 °C. The solution was concentrated *in vacuo* to give a yellow oil and the crude compound purified by reverse phase HPLC (0.2% TFA and a 11-16% acetonitrile gradient on C18 preparative column), yielding 67% TraNP1-SS (white solid). The synthesis of the TraNP1-SS isomer followed a similar procedure, 6 mL of 1 M HCl solution containing 0.2 g (0.26 mmol) of compound 4 (***SS***) was heated up to 50 °C for 8 h to yield 0.12 g of TraNP1-SS (yield 62%). RR-isomer $[\alpha]_D^{20}$= 16.6 (C= 5

mg/mL, methanol), SS-isomer $[\alpha]_D^{20}$= -17.8 (C = 10 mg/mL, methanol); TraNP1-RR ^{1}H NMR (500 MHz, CD_3OD, 333 K): δ = 3.09-3.19 (br, 8H, CH_2N), 3.37-3.38 (t, 4H, $^2J_{H,H}$ = 5 Hz, CH_2SO_2), 3.42 (s, 6H, CH_3S), 3.50-3.55 (br, 14H), 3.58-3.65 (br, 8H), 4.69-4.72 (q, 2H, $^2J_{H,H}$ = 5 Hz); ^{13}C NMR (500 MHz, CD_3OD, 333K): δ = 36.45 (CH_2SO_2), 40.37 ($CH_2CH_2SO_2$) 50.74 (CH_2N), 52.90 (CH_2N), 50.97 (CH_3S), 56.09 (CH_2CONH), 57.64 (CH_2CHO) 67.30 (CHOH) 172.53 (COOH), 173.92 (CONH); FTIR (cm^{-1}) RR-isomer: 3078.0 (w), 2927.7 (w), 1659.9 (s), 1559.7 (w), 1312.2 (s), 1197.7 (s), 1127.6 (vs), 1033.1 (w), 958.7 (w), 831.4 (w), 799.9 (w), 747.0 (w), 719.8 (w); the spectroscopic data of TraNP1-SS are in agreement with its enantiomer. SS isomer HR-MS (ESI): m/z 739.2154 $[M+H]^+$, calcd $[C_{24}H_{46}N_6O_{12}S_4]$ 739.2050. RR isomer HR-MS: m/z 739.2141 $[M+H]^+$, calcd $[C_{24}H_{46}N_6O_{12}S_4]$ 739.2050.

S,S'-(((2,2'-(1,4,7,10-tetraazacyclododecane-1,7 diyl)bis(acetyl))bis(azanediyl))bis(ethane-2,1-diyl))dimethanesulfonothioate, TraNP5

Compound **5** (0.5 g, 0.66 mmol) was deprotected with a mixture of DCM:TFA (*v:v*, 2:3) for 6 h at r.t., then concentrated under reduced pressure and co-evaporated with toluene. The crude product purified by gel filtration. ^{1}H NMR (500 MHz, CD_3OD, 298 K): δ = 2.99-3.01 (br, 8H, CH_2N), 3.16-3.18 (t, 8H, $^2J_{H,H}$ =5 Hz, CH_2N), 3.35-3.38 (t, 4H, $^2J_{H,H}$ = 5 Hz, CH_2SO_2S), 3.45-3.46 (s, 6H, CH_3S), 3.45-3.46 (s, 4H, CH_2NHCO) 3.58-3.61 (t, 4H, $^2J_{H,H}$ = 5 Hz, $CH_2CH_2SO_2S$). ^{13}C NMR (500 MHz, CD_3OD, 298K): δ = 36.42 (CH_2SO_2S), 40.28 ($CH_2CH_2SO_2S$), 44.57 (CH_2N), 50.71 (CH_2N), 51.47 (CH_3S), 57.03 (CH_2CONH), 173.60 (CONH). FTIR (cm^{-1}): 3076.7 (w), 2925.4 (w), 1671.3 (s), 1541.1 (w), 1319.6 (s), 1199.13 (s), 1177.7 (s), 1127.6 (vs), 958.7 (w), 831.4 (w), 798.5 (w), 748.4 (w), 721.2 (w). HR-MS (ESI) SS-isomer: m/z 563.1805 $[M+H]^+$, calcd $[C_{18}H_{38}N_6O_6S_4]$ 563.1814.

(*S*)-3-(4,10-bis(2-((2-((methylsulfonyl)thio)ethyl)amino)-2-oxoethyl)-1,4,7,10-tetraazacyclododecan-1-yl)-2-hydroxypropanoic acid, TraNP3

To a solution of TraNP5 (0.2 g, 0.36 mmol) in methanol (3 mL), methyl (*S*)-oxirane-2-carboxylate (31 μL, 0.36 mmol) was added, the mixture stirred at r.t. for 36 h, and concentrated under reduced pressure. Without purification, the crude mixture was deprotected with a 1 M HCl (2 mL) solution at 50 °C and purified by reverse phase HPLC (0.2% TFA and a 15-20% acetonitrile gradient on C18 preparative column). $[\alpha]_D^{20}$ = -9.1 (C = 2 mg/mL, methanol); ^{1}H NMR (500 MHz, CD_3OD, 333 K): δ = 3.03 (t, 4H, $^2J_{H,H}$ = 5Hz, CH_2N), 3.11 (t, 4H, $^2J_{H,H}$ = 5 Hz, CH_2N), 3.22 (t, 4H, $^2J_{H,H}$ = 5 Hz, CH_2N), 3.38 (t, 4H, $^2J_{H,H}$ = 5 Hz, CH_2SO_2S), 3.43 (s, 6H, CH_3S), 3.43-3.45 (br, 4H, CH_2N, CH_2NHCO), 3.47 (d, 4H, CH_2NH), 3.51-3.56 (m, 2H, CH_2CHOH), 3.59-3.62 (t, 4H, $^2J_{H,H}$ = 5 Hz), 4.53 (m, 1H, CHOHCOOH); ^{13}C NMR (500 MHz, CD_3OD, 343K): δ = 36.47 (CH_2SO_2S), 40.30 ($CH_2CH_2SO_2S$), 44.65 (CH_2N), 50.85 (CH_2N), 50.16 (CH_2N), 56.96 (CH_2NH), 50.89 (CH_3S), 53.73 (CH_2CONH), 58.03 (CH_2CHOH), 67.73 ($CHOHCH_2$), 173.30 (COOH), 174.87 (CONH); FTIR (cm^{-1}): 3065.0 (w), 2884.3 (w), 1669.9 (s), 1558.3 (w), 1456.7 (w), 1418.1 (w), 1313.6 (m), 1200.6 (s), 1130.5 (vs), 958.7 (w), 832.8 (w), 799.9 (w), 748.4 (w), 712.2 (w); HR-MS (ESI): m/z 651.1973 $[M+H]^+$, calcd [$C_{21}H_{42}N_6O_9S_4$] 651.1896.

Metal complex: To a solution of TraNP1-SS (20.7 mg, 28 μmol) in 280 μL ACN, 1.1 equivalent $CoCl_2 \cdot 6H_2O$ was added. The mixture was stirred at r.t. for 3 h and the formation of metal complex was checked by LC/MS. Without further purification, Co-TraNP1-SS was used to protein sample labeling. The other metal ions Zn(II) and Mn(II) were chelated to TraNPs following the same procedure. Co-TraNP1-SS HR-MS: m/z 796.1319 $[M+H]^+$, calcd [$C_{24}H_{44}CoN_6O_{12}S_4$] 796.1232; Zn-TraNP1-SS HR-MS: m/z 401.0667 $[M+H]^{2+}$, calcd [$C_{24}H_{44}ZnN_6O_{12}S_4$] 401.0596; Mn-TraNP1-SS HR-MS: m/z 792.1354 $[M+H]^+$, calcd [$C_{24}H_{44}MnN_6O_{12}S_4$] 791.1282; Co-TraNP1-RR HR-MS: m/z 796.1317 $[M+H]^+$, calcd [$C_{24}H_{44}CoN_6O_{12}S_4$] 796.1232; Zn-TraNP1-RR HR-MS: m/z 401.0687 $[M+2H]^{2+}$ calcd [$C_{24}H_{44}ZnN_6O_{12}S_4$] 401.0669. Co-TraNP3-S HR-MS: m/z 708.1149 $[M+H]^+$, calcd [$C_{24}H_{44}CoN_6O_{12}S_4$] 708.1150.

Protein labeling: To link TraNPs to ^{15}N labelled T4Lys, BCX or ubiquitin, produced as described previously,[3] the protein sample (1 mL, 200−400 μM) was treated with DTT (final concentration 10 mM) at 0 °C. After 1 h, the protein solution was loaded on a PD-10 column (GE Healthcare), pre-equilibrated with labeling buffer (20 mM sodium phosphate, 150 mM NaCl, pH 7.0, argon degassed) to remove DTT. The eluted protein was added to a solution of labeling buffer containing TraNP (6 eq) under an argon atmosphere. The mixture was stirred at 4 °C for 8 h. Then, the mixture was concentrated to 500 μL and purified by using a Superdex 75 gel filtration column. *Tag 1* attachment to ubiquitin and T4Lys followed the procedure described before.[4] Briefly, the protein solution (200 μM) was incubated with 10 mM of DTT at 4 ºC for 1 h. The PD10 column was equilibrated with labelling buffer (50 mM HEPES pH 7.5 for ubiquitin, 20 mM sodium phosphate, 150 mM NaCl pH 7.5 for T4Lys, argon degassed) and the protein solution was loaded to remove the DTT and the eluate was added to 10 eq. *tag 1* solution in labelling buffer to react at r.t. for 2 h. The labeled proteins were concentrated to 500 μL and purified by using a Superdex 75 gel filtration column. The yield of labeling estimated from the LC/MS and NMR, was more than 95%. The purified *tag 1*-ubiquitin and *tag 1*-T4Lys (120 μM) were treated with 1 mM EDTA and purified by PD10 column, before incubate with $CoCl_2 \cdot 6H_2O$ (1.2 equivalent or 10 equivalent) and ZnI_2 (1.2 equivalent) at 4 ºC pH 7.5, for 12 h, respectively. The stock solutions containing 10 mM of the metal salts in the same buffer as the protein solution.

Protein NMR spectroscopy: The NMR samples of T4Lys-TraNP (100−200 μM) were prepared in 30 mM sodium phosphate pH 5.5 buffer containg 100 mM NaCl and 6% (*v/v*) D_2O. The NMR samples of BCX-TraNP (100−200 μM) contained 25 mM sodium acetate buffer pH 5.5, 6% (*v/v*) D_2O. For ubiquitin NMR samples, a buffer of 50 mM HEPES pH 7.5, 6% (*v/v*) D_2O was used. All ^{1}H-^{15}N HSQC were recorded on a Bruker Avance III 600 MHz (14 T) spectrometer, at 298 K for T4Lys/ubiquitin-TraNP/*tag 1* samples and 293 K for BCX-TraNP samples. Data were processed with Topspin 3.5 and analyzed with CcpNmr Analysis version 2.4.0.[5] T4Lys and BCX resonance

assignments were kindly provided by Simon P. Skinner and Fredj Ben Bdira, respectively, based on previous work[6]. Ubiquitin resonance assignments were kindly provided by Carlos A. Castañeda and David Fushman.

PCS data analysis: The Δχ tensors were calculated using Numbat software.[7] Published structures of T4Lys, BCX and ubiquitin (PDB entries 2lzm, 2bvv and 2mjb)[8], to which hydrogens had been added, were used. The experimental PCS were fitted to equation 1:

$$\text{PCS} = \frac{1}{12\pi r^3}\left[\Delta\chi_{ax}(3\cos\theta^2 - 1) + \frac{3}{2}(\Delta\chi_{rh}(\sin\theta^2\, cos2\Omega)\right] \qquad 1$$

where r, θ, and Ω are the polar coordinates of the nucleus with respect to the principle axes of the Δχ-tensor and $\Delta\chi_{ax}$ and $\Delta\chi_{rh}$ are the axial and rhombic components of the Δχ tensor, respectively, by optimizing the position of the metal, the Euler angles describing the tensor frame orientation and the sizes of $\Delta\chi_{ax}$ and $\Delta\chi_{rh}$, which constitutes an eight-parameter fit. All the experimental PCS values are given in Appendix.

The Q_a factor provides a normalized measure for the agreement between a set of observed and calculated data according to equation 1:[9]

$$Q_a = \sqrt{\frac{\Sigma(\delta_{PCS,i}^{cal} - \delta_{PCS,i}^{exp})^2}{\Sigma(\,|\delta_{PCS,i}^{cal}\,| + |\delta_{PCS,i}^{exp}\,|)^2}} \qquad 2$$

PRE Data Analysis: The intensity ratio of amide resonances in the paramagnetic sample spectra (Mn(II)-TraNP1-T4Lys, I_{para}) and diamagnetic sample spectra (Zn(II)-TraNP1-T4Lys, I_{dia}) was determined and normalized, as described previously.[10] The $R_{2,para}$ was calculated using equation 2, where the $R_{2,dia}$ was obtained from the width at half-height in Hz ($\Delta\nu_{1/2}$) of a Lorentzian fit in the proton dimension by using $R_2 = \pi\Delta\nu_{1/2}$ in the diamagnetic spectra, and t is the total INEPT evolution time of the HSQC (9 ms).[10a] The standard deviation of the spectrum noise level was determined by

CcpNmr.[5] The error of $R_{2,para}$ was calculated as described.[10b] The H^N-to-metal distance (r) was calculated with equation 3 [9] where τ_c is the rotational correlation time, which was calculated by HYDRONMR as described before (8 ns for T4Lys),[11] γ_I is the gyromagnetic ratio of nucleus I, g_e is the electron g-factor, β is the Bohr magneton, S is the total electron spin quantum number, μ_0 is the permeability of vacuum and ω_I the Larmor frequency of the nucleus.

$$\frac{I_{para}}{I_{dia}} = \frac{R_{2,dia}e^{(-R_{2,para}t)}}{R_{2,dia}+R_{2,para}} \qquad 3$$

$$r = \sqrt[6]{\frac{1}{R_{2,para}}\frac{\gamma_I^2 g_e^2 \beta^2 \mu_0^2 (S+1)S}{240\pi^2}\left(4\tau_c + \frac{3\tau_c}{1+\omega_I^2\tau_c^2}\right)} \qquad 4$$

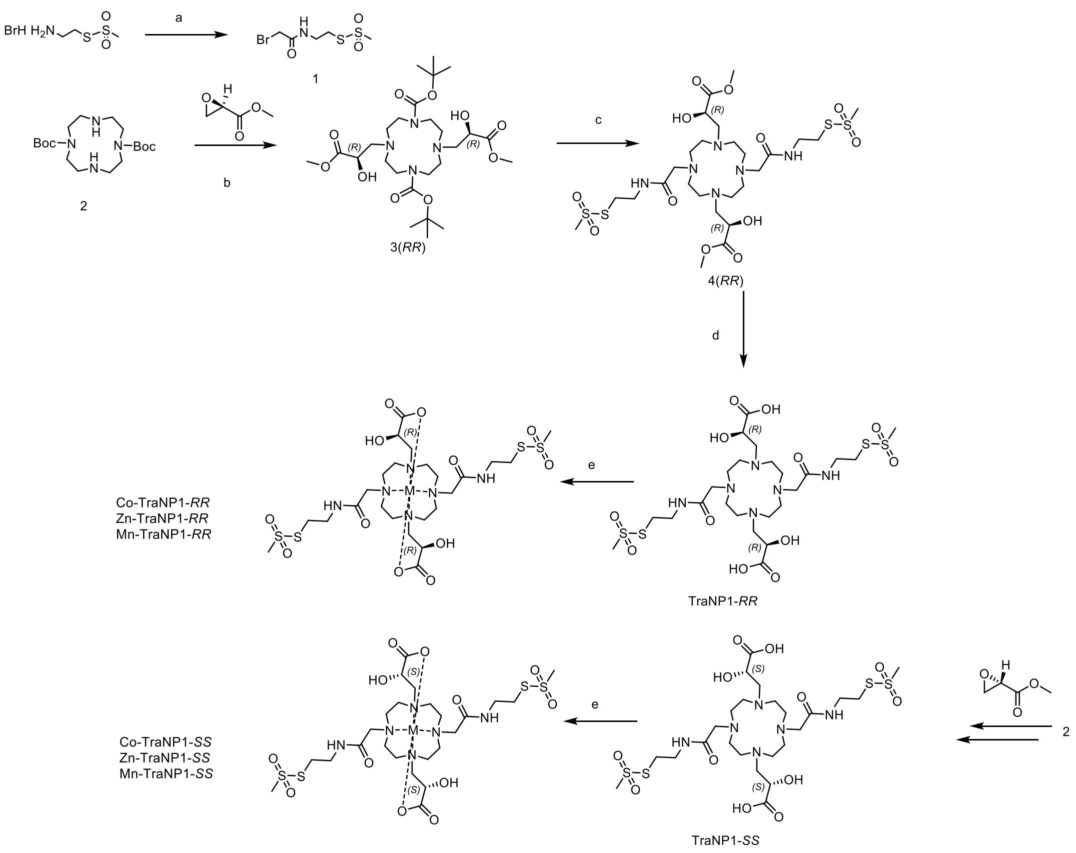

Scheme S1. Synthesis of TraNP1. a) Acetyl bromide, K_2CO_3, H_2O, 0 °C; b) Methyl (2S)-glycidate, MeOH r.t., 48 h; c) i) TFA: DCM (v/v) 4:1, r.t., 16 h; ii) compound 1, K_2CO_3, ACN, r.t., 12 h; d) 1 M HCl, 50 °C, 4 h; e) ACN, r.t., 3 h.

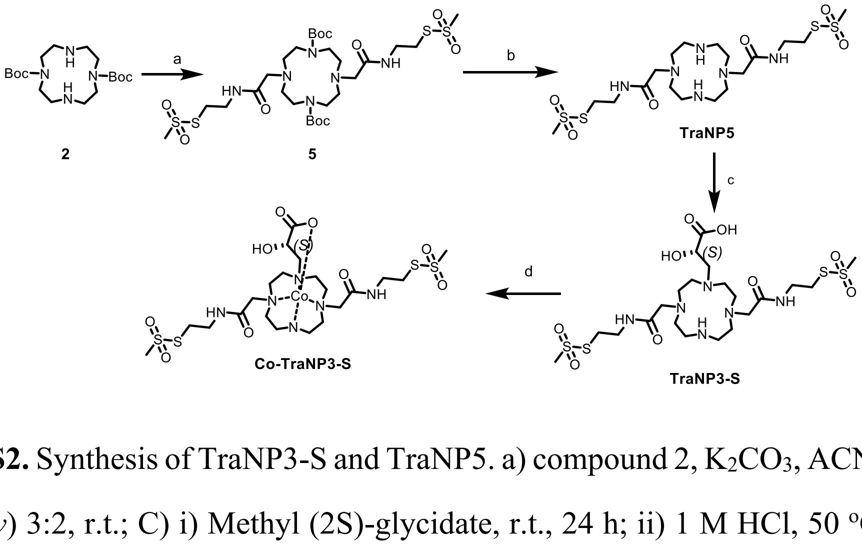

Scheme S2. Synthesis of TraNP3-S and TraNP5. a) compound 2, K_2CO_3, ACN; b) TFA: DCM (*v/v*) 3:2, r.t.; C) i) Methyl (2S)-glycidate, r.t., 24 h; ii) 1 M HCl, 50 °C, 4 h; d) $CoCl_2 \cdot 6H_2O$, ACN, r.t., 3 h.

Table S1. PCS-based $\Delta\chi$-tensor parameters of Co(II)-TraNP1 attached to T4Lys K147C/T151C, fitted to three crystal structures.

PDB	2lzm [8a]		3dke [12]		1l68 [13]	
Probes	**RR**	**SS**	**RR**	**SS**	**RR**	**SS**
$\Delta\chi_{ax}$ [a]	5.2±0.1	4.6±0.1	5.2±0.2	4.7±0.1	5.2±0.2	4.6±0.1
$\Delta\chi_{rh}$ [a]	1.2±0.1	0.9±0.1	1.3±0.1	0.9±0.2	1.3±0.1	0.9±0.2
X (Å) [b]	3.4±0.1	3.9±0.1	43.7±0.2	44.2±0.2	43.9±0.1	44.4±0.1
Y (Å) [b]	-3.6±0.1	-4.3±0.1	8.3±0.2	7.5±0.2	8.8±0.1	8.0±0.2
Z (Å) [b]	-4.4±0.1	-4.2±0.1	-8.9±0.1	-8.6±0.1	-9.1±0.1	-9.0±0.1
α (°)	57±1	57±4	56±1	56±5	58±1	58±1
β (°)	68±1	69±1	68±1	69±2	68±1	69±2
γ (°)	118±1	103±3	119±1	105±4	118±1	103±1
Restraints	81	89	78	84	78	89
Q_a	0.04	0.04	0.04	0.04	0.04	0.04

[a] $\Delta\chi_{ax}$ and $\Delta\chi_{rh}$ are in 10^{-32} m^3; [b] the coordinates of the Co(II) are in the frame defined by the PDB file;

Table S2. PCS-based $\Delta\chi$-tensor parameters of Co(II)-TraNP1 attached to T109C/T111C BCX, fitted to three crystal structures.

PDB	2bvv[8b]		3vzm[14]		1bvv[8b]	
Probes	**RR**	**SS**	**RR**	**SS**	**RR**	**SS**
$\Delta\chi_{ax}$ [a]	3.8±0.2	2.6±0.1	3.8±0.2	2.6±0.1	3.8±0.2	2.6±0.1
$\Delta\chi_{rh}$ [a]	0.5±0.1	0.6±0.1	0.5±0.1	0.6±0.2	0.5±0.1	0.6±0.1
X (Å) [b]	17.3±0.2	17.1±0.1	17.2±0.2	17.4±0.1	17.4±0.2	17.1±0.1
Y (Å) [b]	13.9±0.2	14.1±0.2	13.8±0.2	14.1±0.2	13.6±0.2	14.5±0.2
Z (Å) [b]	39.3±0.1	39.4±0.1	39.6±0.1	39.4±0.1	39.2±0.1	39.7±0.2
α (°)	2.7±1	173±2	4.5±4	169±3	177±4	176±2
β (°)	18±2	156±1	18±2	155±1	161±2	157±1
γ (°)	137±1	37±4	134±1	31±1	38±1	41±1
Restraint	105	100	105	99	104	99
Q_a	0.05	0.06	0.05	0.06	0.05	0.07

[a] $\Delta\chi_{ax}$ and $\Delta\chi_{rh}$ are in 10^{-32} m^3; [b] the coordinates of the Co(II) are in the frame defined by the PDB file.

Table S3. PCS-based $\Delta\chi$-tensor parameters of Co(II)-TraNP1-SS attached to E24C/A28C ubiquitin, fitted to three crystal structures.

PDB	2mjb[8c]	1d3z[15]	3ons[16]
$\Delta\chi_{ax}$ [a]	2.0±0.1	2.0±0.1	1.8±0.1
$\Delta\chi_{rh}$ [a]	0.4±0.1	0.6±0.1	0.3±0.1
X (Å) [b]	4.2±0.6	5±0.6	3.9±0.4
Y (Å) [b]	3.6±0.3	4.2±0.5	2.4±0.4
Z (Å) [b]	-14.4±0.3	-14.1±0.4	-14.1±0.2
α (°)	71±16	71±12	73±22
β (°)	56±2	50±4	65±2
γ (°)	96±13	88±11	93±20
Restraint	45	45	45
Q_a	0.04	0.05	0.04

[a] $\Delta\chi_{ax}$ and $\Delta\chi_{rh}$ are in 10^{-32} m^3; [b] the coordinates of the Co(II) are in the frame defined by the PDB file.

Table S4. PCS-based $\Delta\chi$ tensor parameters of *tag 1* and TraNP1-SS attached to ubiquitin E24C/A28C (PDB entry 2mjb)[8c] and T4Lys K147C/T151C (PDB entry 2lzm)[8a].

Protein	Ubiquitin			T4Lys	
Probes	***Tag 1*** [b]	***Tag 1*** [c]	**TraNP1-SS**	***Tag 1***	**TraNP1-SS**
$\Delta\chi_{ax}$ [a]	-7.4±0.04	-7.2±0.1	2.2±0.1	-6.8±0.2	4.6±0.1
$\Delta\chi_{rh}$ [a]	-1.4±0.04	-1.3±0.1	0.2±0.1	-0.46±0.1	0.9±0.1
Restraints	66	45	43	53	89
Q_a	0.02	0.02	0.04	0.04	0.04
Co(II)-Cys C_α distance (Å)	7.0 (C24)<br>7.4 (C28)	8.0 (C24)<br>7.4 (C28)	9.0 (C24)<br>8.8 (C28)	8.0 (C147)<br>7.0 (C151)	7.9 (C147)<br>8.3 (C151)

[a] in 10^{-32} m^3; [b] Tensor parameters based on PCS reported in [4]; [c] Tensor parameters based on work performed in this study.

Table S5. Predicted PCS changes for some paramagnetic metals upon small changes in the position of the nucleus relative to the Δχ tensor frame. The absolute change in PCS is given starting with a PCS of 0.40 ppm upon (I) moving the nucleus by 1 Å over the sphere with radius r or (II) by reducing the vector r by 1 Å. Also the distance from the metal above which the PRE for a ^{1}H nucleus is less than 20 Hz is given. The values $\Delta\chi_{ax}$ and $\Delta\chi_{rh}$ are examples.

Metal	$\Delta\chi_{ax}$ [d]	$\Delta\chi_{rh}$ [d]	Distance r[a]	1 Å move at given distance[b]	r - 1 Å	PRE [c] region
				ΔPCS (%)[e]	ΔPCS (%)[e]	
**Tm(III)**	55.0	6.0	36.10	0.021 (5.3%)	0.035 (9%)	14.1
**Yb(III)**	8.5	2.5	19.75	0.034 (8.5%)	0.067 (17%)	10.2
**Pr(III)**	3.5	0.6	14.50	0.054 (13.5%)	0.096 (24%)	8.7
**Co(II)**	3.5	0.6	14.50	0.054 (13.5%)	0.096 (24%)	9.3

[a] A PCS of 0.40 ppm is obtained at this distance and polar coordinates θ = 30°, Ω = 0°, r is in Å. [b] Direction of the move is a rotation along the χ_{yy} axis, increasing only θ. [c] At 20 T (850 MHz), τ_c = 15 ns assuming that the PRE is the sum of Solomon and Curie contributions. The latter dominates in all cases. PRE region is in Å. [d] $\Delta\chi_{ax}$ and $\Delta\chi_{rh}$ are in 10^{-32} m^3. [e]**ΔPCS** is in ppm.

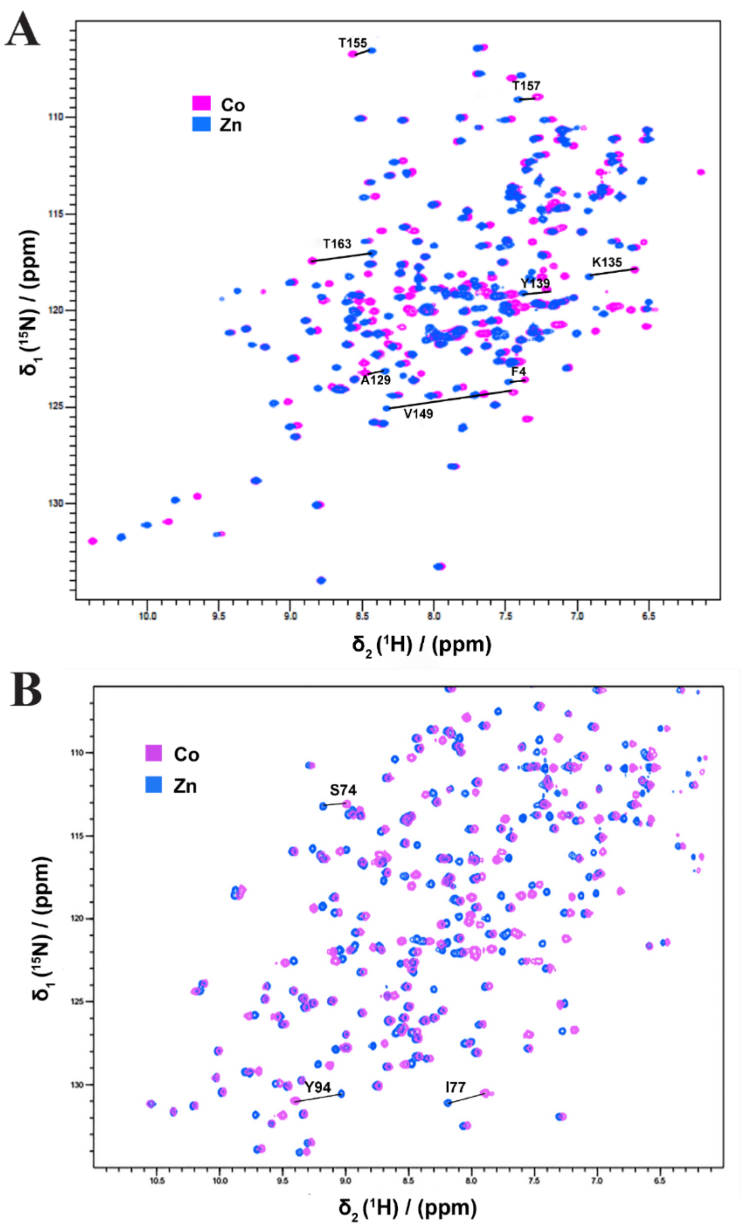

Figure S1. Overlay of ^{1}H-^{15}N HSQC spectra of Co(II) (magenta) and Zn(II) (blue) loaded TraNP1-SS attached to T4Lys K147C/T151C (A) and BCX E78Q/T109C/T111C (B). Several PCS are indicated with solid lines and residue numbers. The NMR spectra were recorded at 14.1 T (600 MHz).

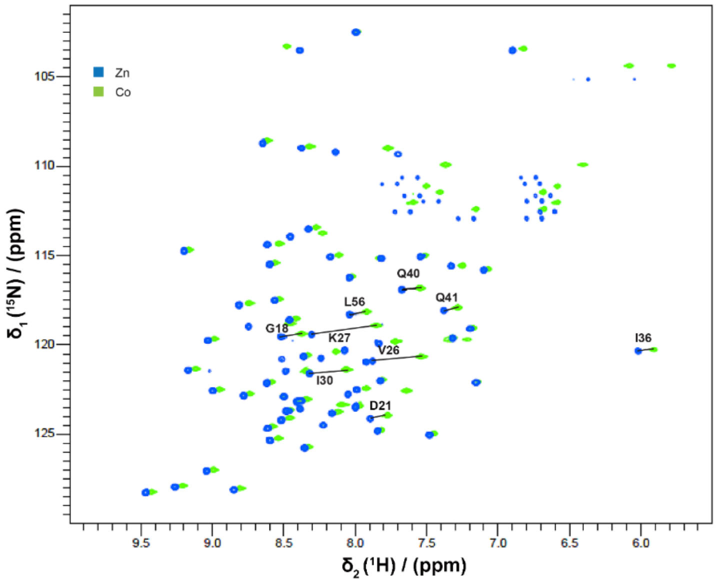

Figure S2. Overlay of ^{1}H-^{15}N heteronuclear correlation spectra of Co(II) (green, HSQC) and Zn(II) (blue, TROSY-HSQC) loaded TraNP1-SS attached to ubiquitin E24C/A28C. Several PCS are indicated with solid lines and residue numbers. The NMR spectra were recorded at 14.1 T (600 MHz).

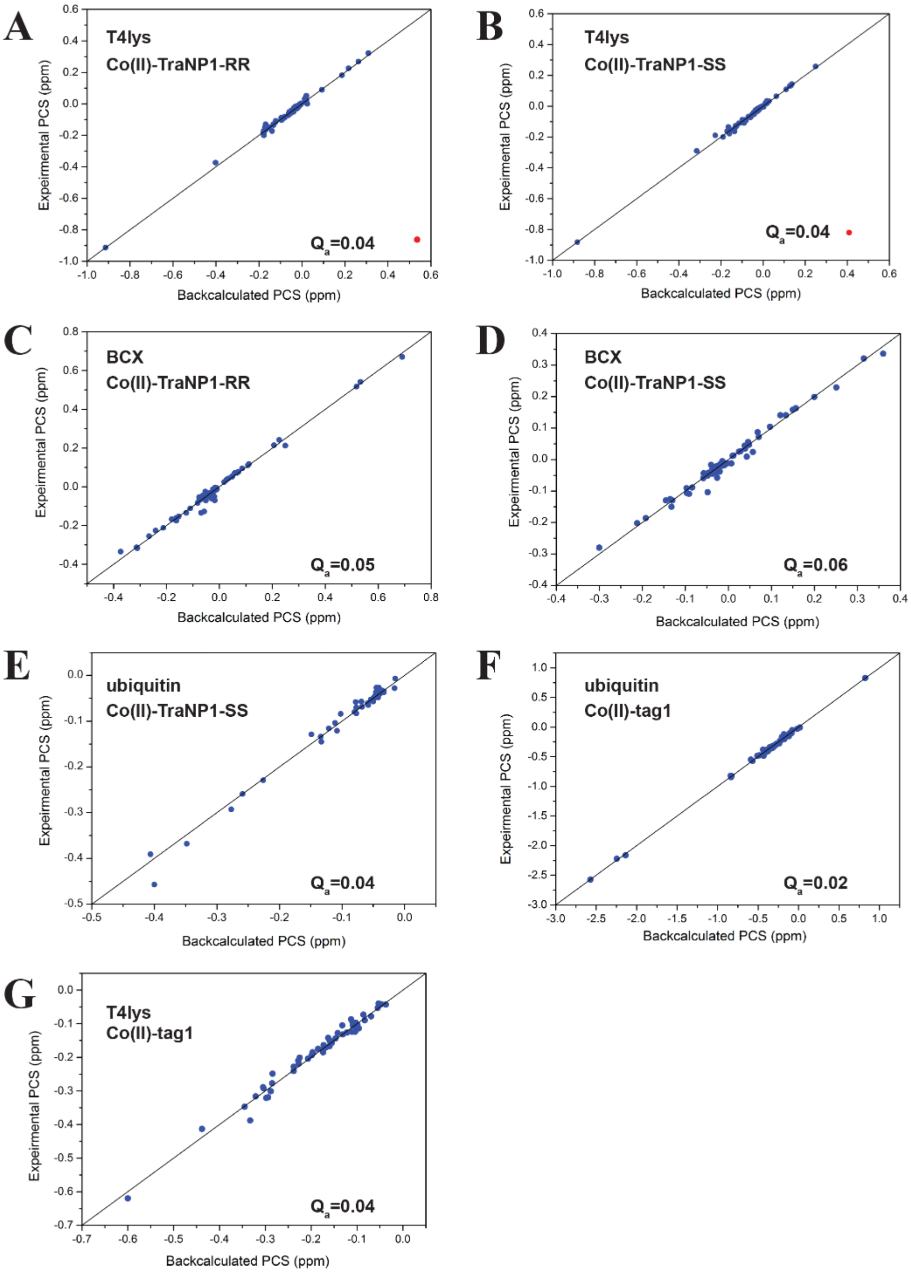

Figure S3. Experimental ^{1}HN PCS (ppm) of Co(II)-TraNP1 were plotted against the back-calculated values after fitting to eq. S1. A) T4Lys K147C/T151C (TraNP1-RR); B) T4Lys K147C/T151C (TraNP1-SS); C) BCX E78Q/T109C/T111C (TraNP1-RR); D) BCX E78Q/T109C/T111C (TraNP1-SS); E) ubiquitin (TraNP1-SS); F) ubiquitin (*tag 1*); G) T4Lys K147C/T151C (*tag 1*). The solid line represent a perfect correlation.

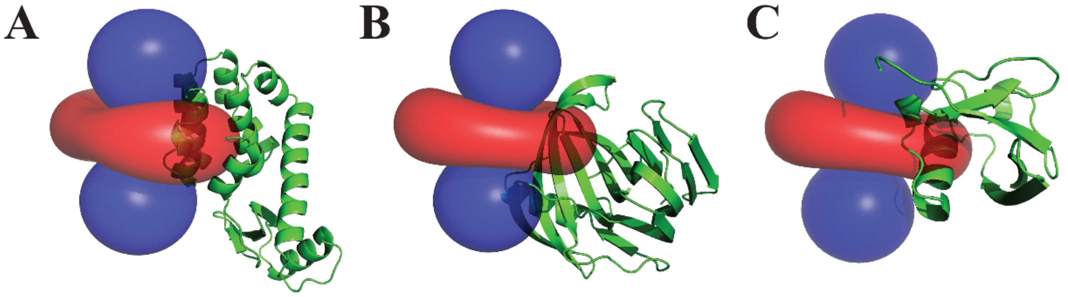

Figure S4. PCS iso-surfaces of TraNP1-SS plotted on the structures of T4Lys K147C/T151C (PDB entry 2zlm)[8a] (A), BCX E78Q/T109C/T111C (PDB entry 2bvv)[8b] (B) and ubiquitin E24C/A28C (PDB entry 2mjb)[8c] (C). The protein backbones are drawn in green ribbon representation. The iso-surfaces correspond to PCS of ±0.2 ppm. Positive and negative PCS was indicated by blue and red, respectively.

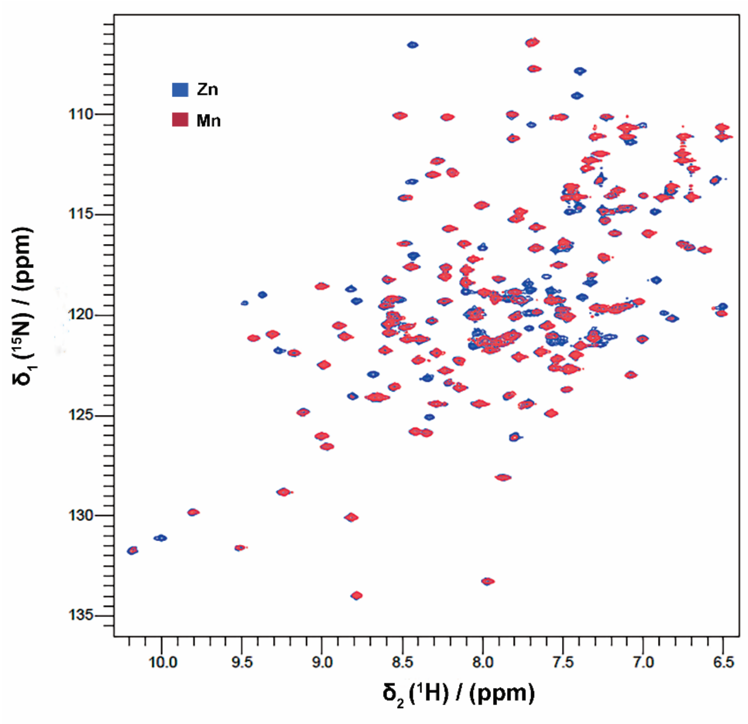

Figure S5. Overlay of ^{1}H-^{15}N HSQC spectra of Mn(II)-TraNP1-SS (red) and Zn(II)-TraNP1-SS (blue) attached to T4Lys K147C/T151C. The NMR spectra were recorded at 14.1 T (600 MHz).

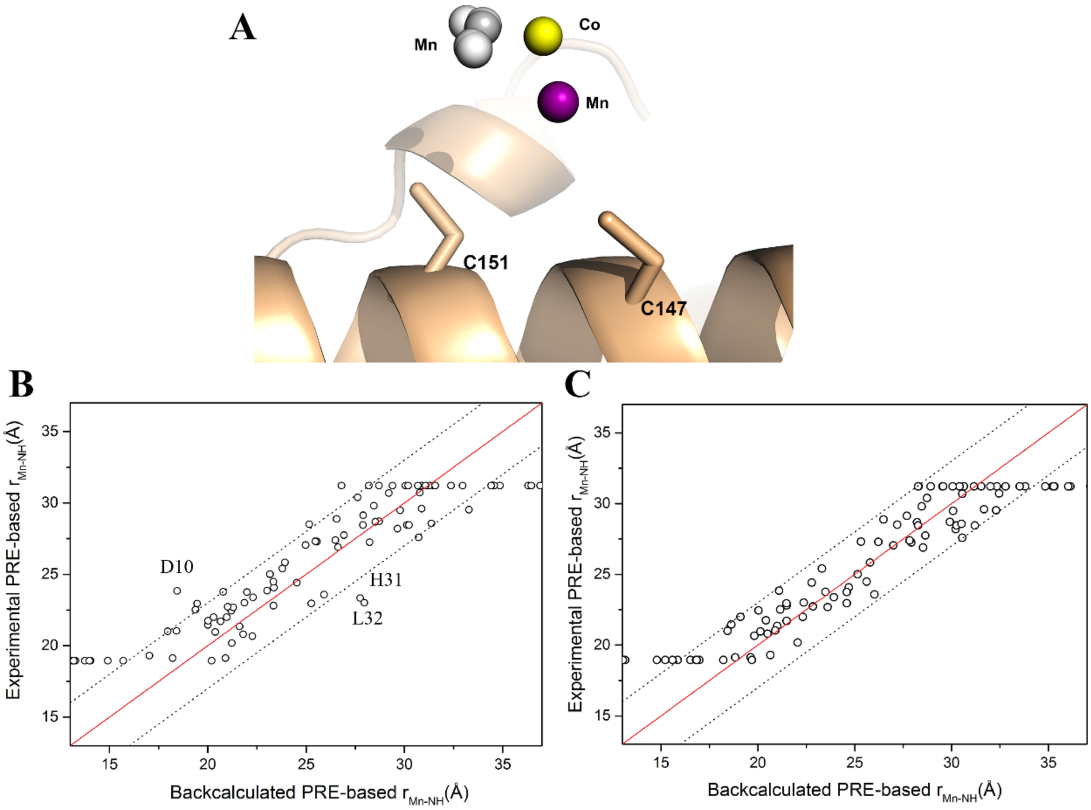

Figure S6. Analysis of PRE data. A) The positions obtained for the metal in TraNP1-SS relative to the T4Lys K147C/T151C backbone (wheat, PDB entry 2lzm[8a]) are shown as spheres. The PCS-based Co(II) position is in yellow. The position of purple sphere is found when including all Mn(II) PRE data. The grey spheres are found when the PRE for residues 10, 31 and 32 are left out, in five fits with random starting positions of the metal; B+C) Experimental amide proton-to-Mn(II) distances plotted against the back-calculated values, including (B) or excluding (C) the distances for residues 10, 31 and 32. The red solid lines represent a perfect correlation and the dotted lines indicate a ±3 Å range.

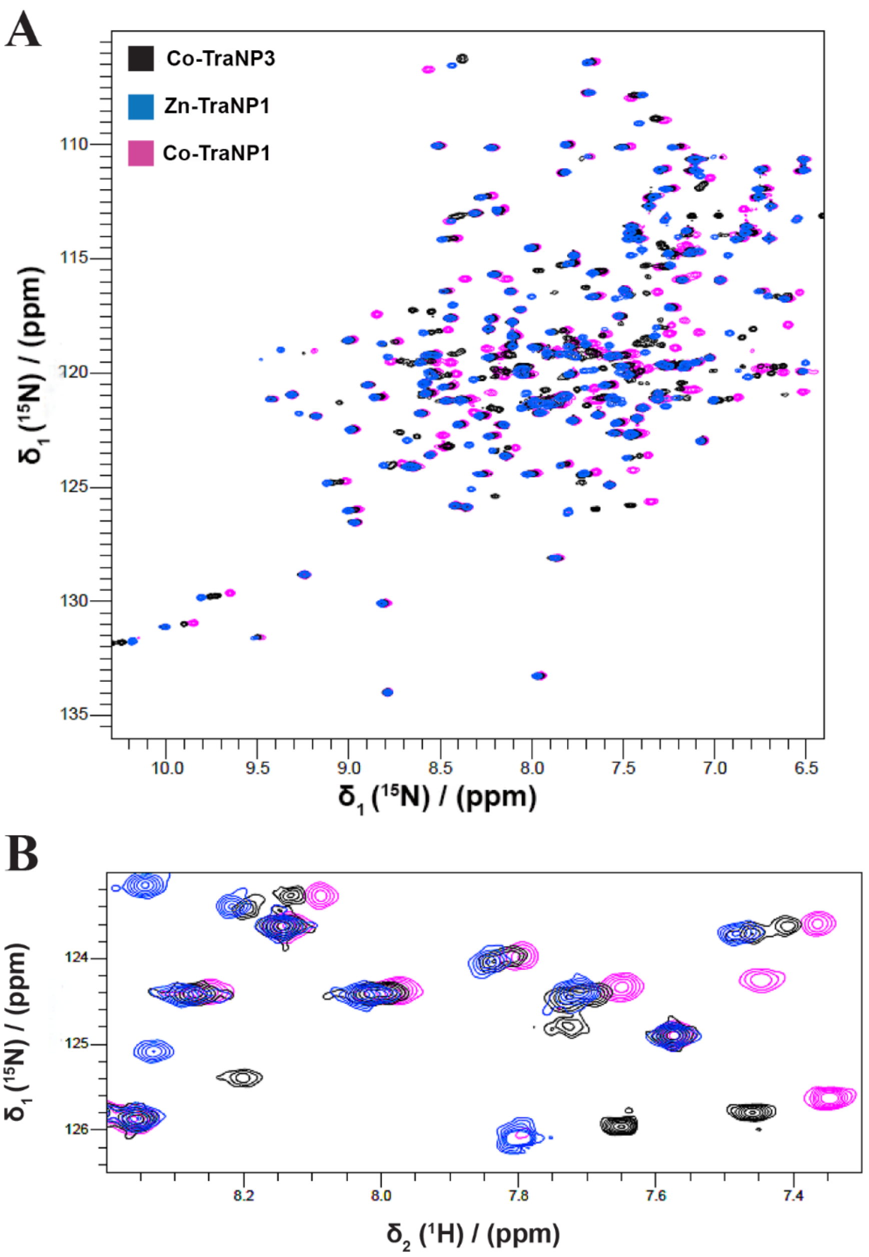

Figure S7. Overlay of ^{1}H-^{15}N HSQC spectra for various TraNPs attached to T4Lys K147C/T151C. A) Full spectrum; B) Detail. The NMR spectra were recorded at 14.1 T (600 MHz).

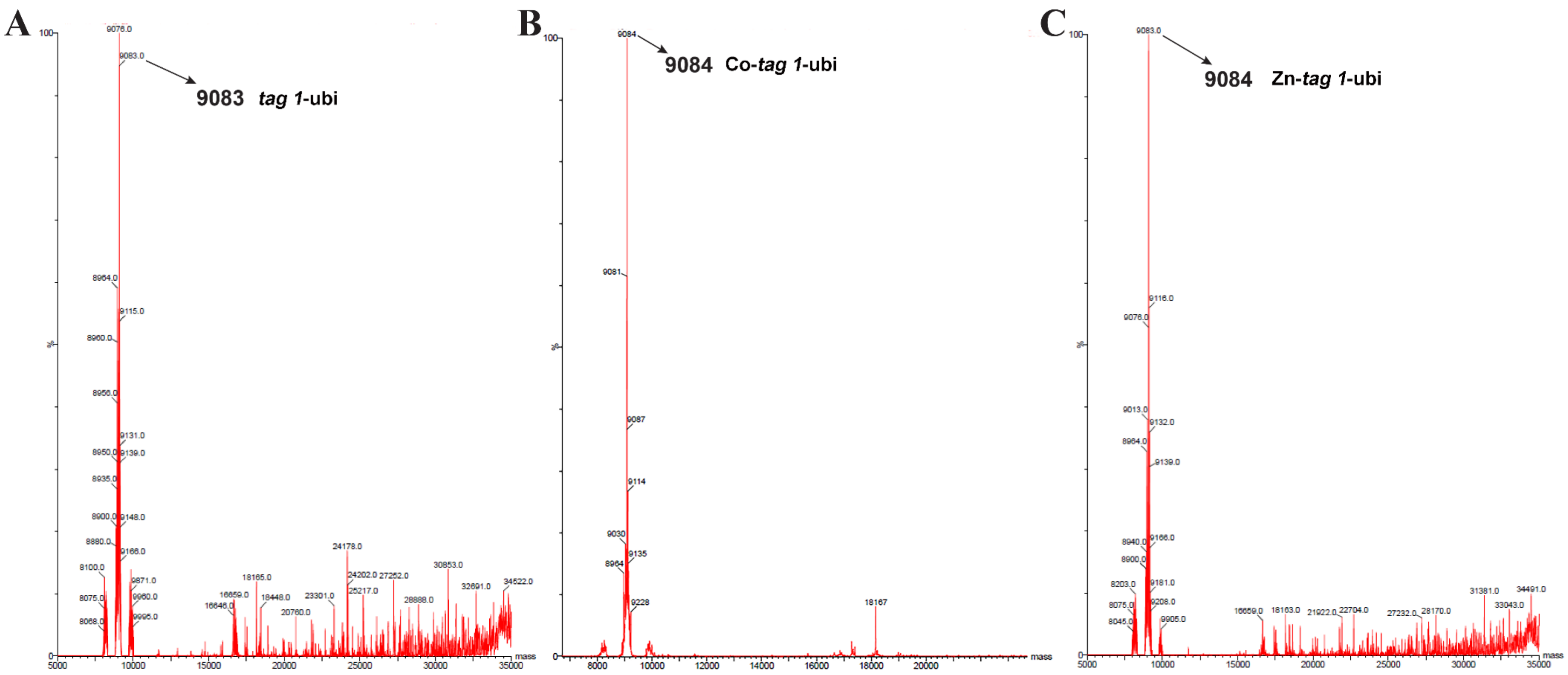

Figure S8. ESI-TOF mass spectra of 15N-enriched E24C/A28C ubiquitin mutant linked to *tag 1* (A), Co-*tag 1* (B) and Zn-*tag 1* (C).

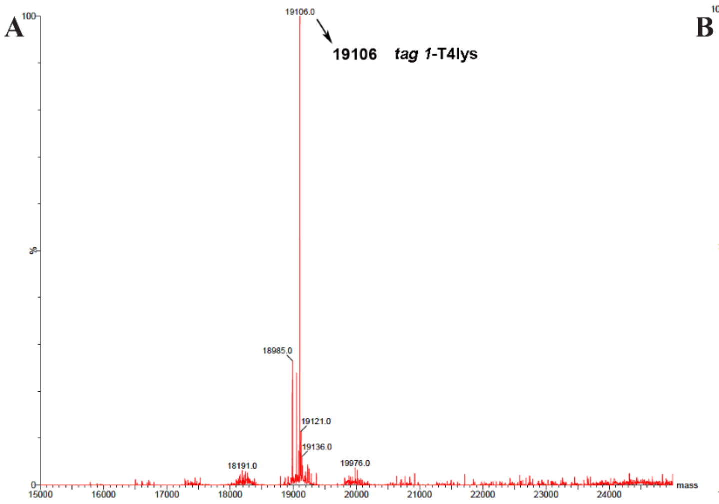

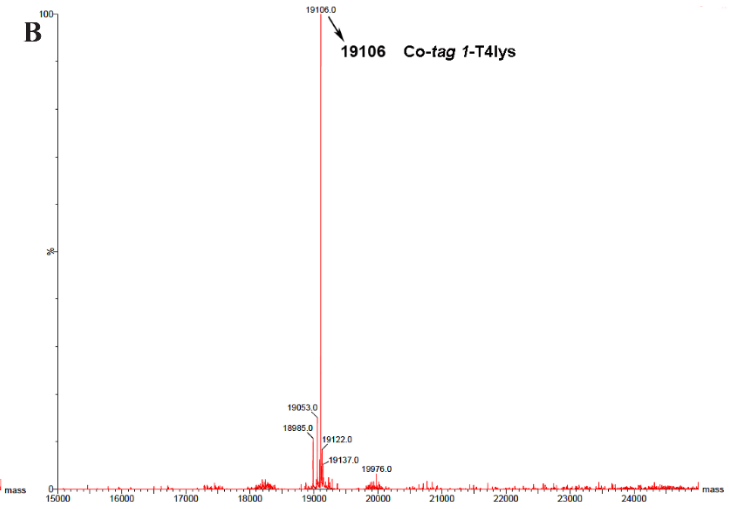

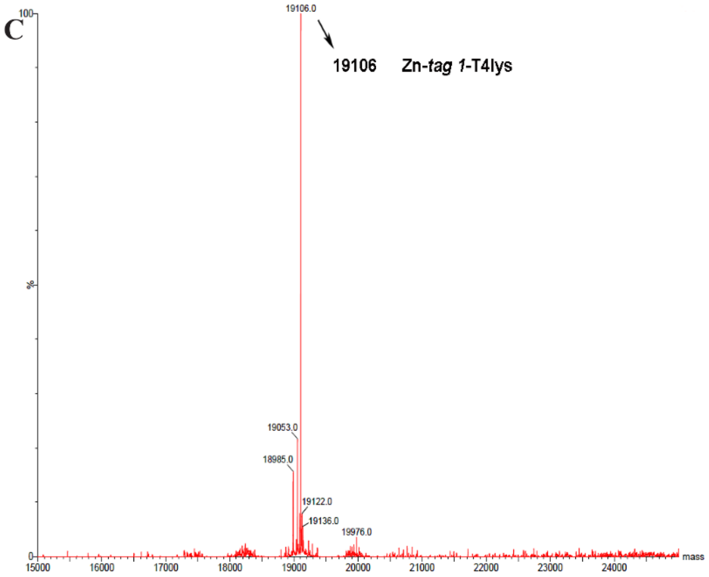

Figure S9. ESI-TOF mass spectra of 15N-enriched T147C/K151C T4Lys mutant linked to *tag 1* (A), Co-*tag 1* (B) and Zn-*tag 1* (C).

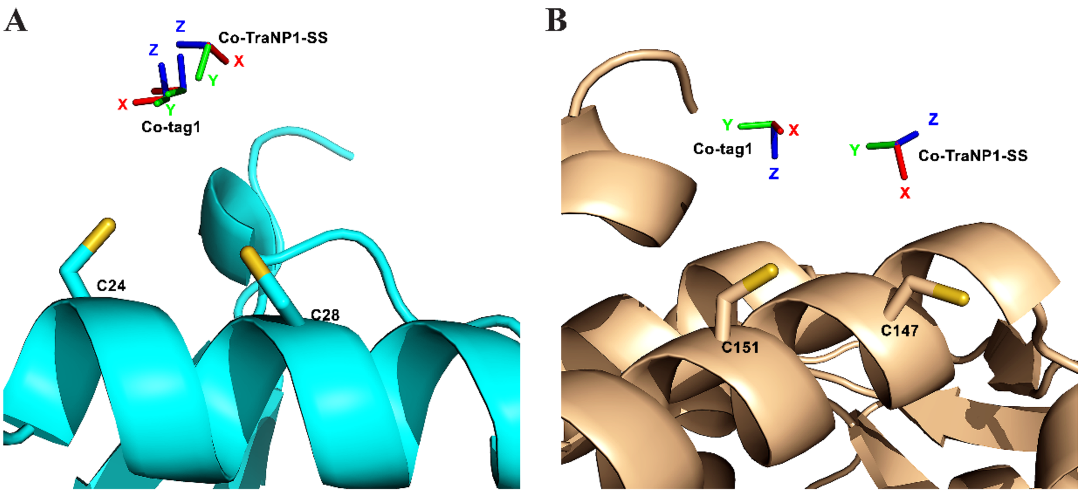

Figure S10. Metal positions and tensor orientations of Co(II)-TraNP1-SS and Co(II)-tag 1 attached to ubiquitin E24C/A28C (A) and T4Lys K147C/T151C (B). In (A) the left tensor of *tag 1* is based on fitting the PCS reported in [4]; the right one is based on the PCS observed in the current work. The proteins are shown in ribbons, the cysteines were modelled into the structure and are shown as sticks.

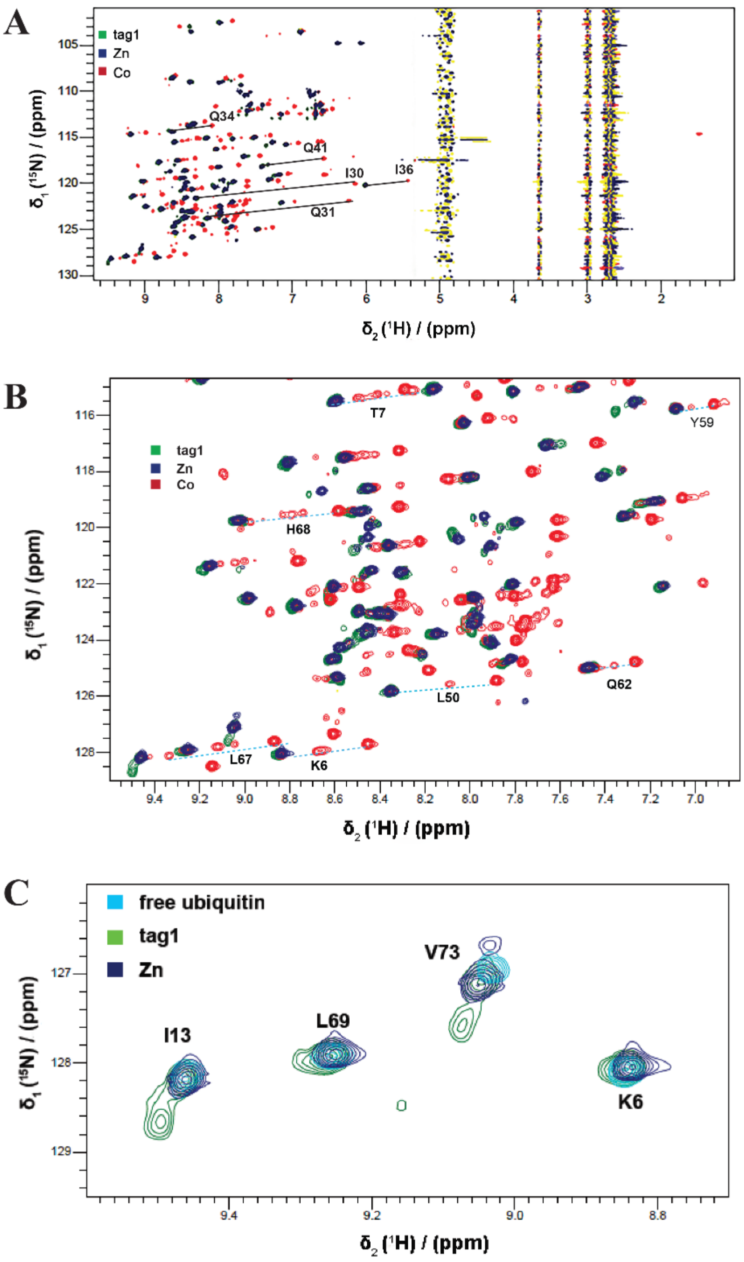

Figure S11. Overlay of ^{1}H-^{15}N HSQC spectra of Co(II)-tag 1 (red), Zn(II)-tag1 (dark blue), metal-free tag 1 (green) linked to ubiquitin E24C/A28C and untagged ubiquitin E24C/A28C (cyan). A) Full spectrum, several PCS are indicated with black solid lines and residue numbers; B) Detail, showing some amides for which multiple PCS are observed (cyan dashed lines); C) Detail, showing the double peaks that appear in ubiquitin linked to *tag 1* but not in the untagged protein or when *tag 1* is bound to Zn(II). The NMR spectra were recorded at 14.1 T (600 MHz).

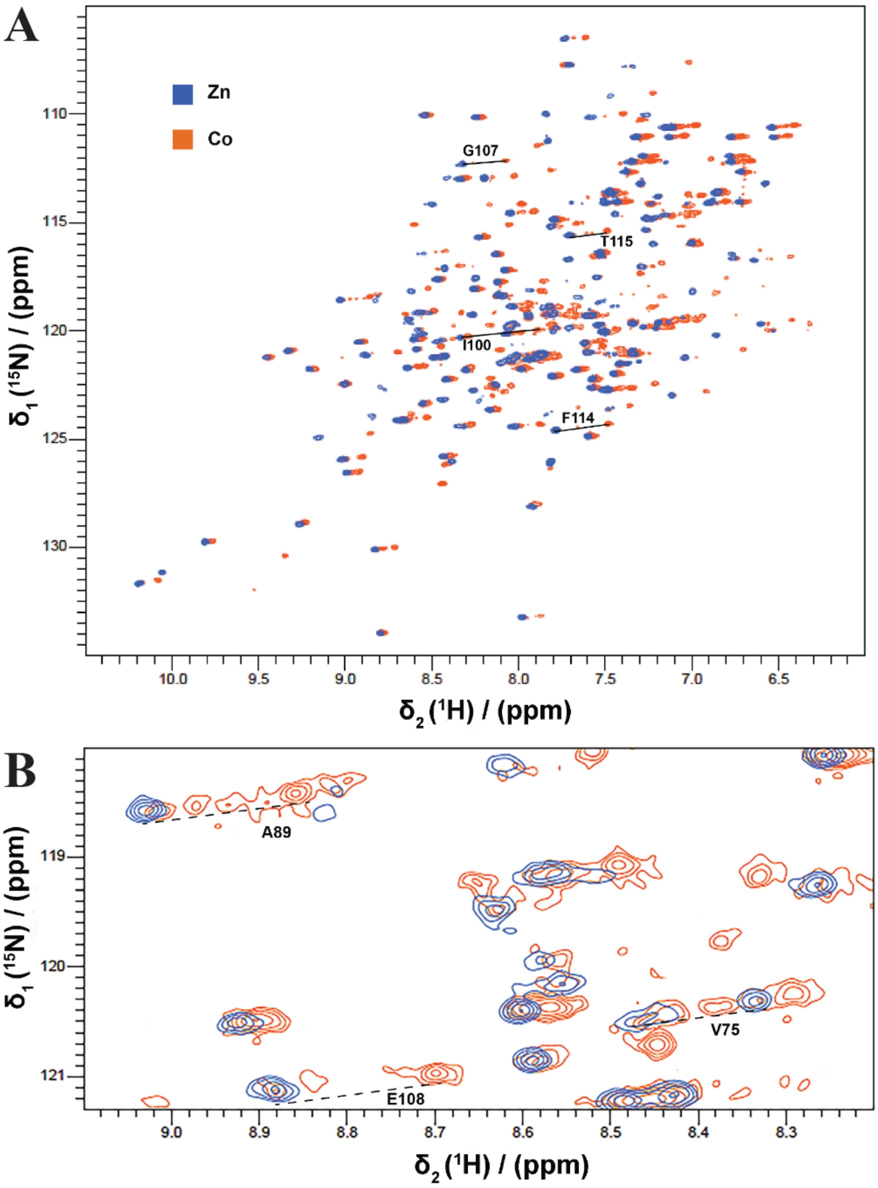

Figure S12. Overlay of ^{1}H-^{15}N HSQC spectra of T4Lys K147C/T151C linked to Co(II) (orange, 10 eq.) or Zn(II) (blue, 1.2 eq.) loaded *tag 1*. A) full spectrum; B) Detail. Several PCS are indicated with solid lines and residue numbers. The NMR spectra were recorded at 14.1 T (600 MHz).

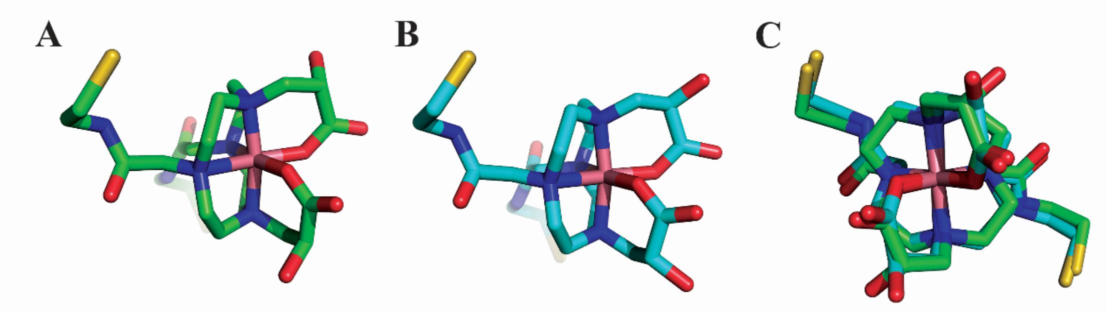

Figure S13. Structure models of TraNP1. A) Co-TraNP1-SS, side view; B) Co-TraNP1-RR, side view; C) overlay of Co-TraNP1-RR and -SS, view along the symmetry axis.

References

[1] a) W.-M. Liu, P. H. Keizers. M. A. S. Hass, A. Blok, M. Timmer, A. J. C. Sarris, M. Overhand, M. Ubbink, *J. Am. Chem. Soc.* **2012**, 17306–17317; b) P. H. J. Keizers, Y. Hiruma, M. Overhand, and M. Ubbink, *J. Am. Chem. Soc.* **2008**, 14802–14812.

[2] P. H. J. Keizers, M. Overhand, M. Ubbink, *J. Am. Chem. Soc.* **2007**, 9292-9293.

[3] a) C. A. Castañeda, L. Spasser, S. N. Bavikar, A. Brik, D. Fushman, *Angew. Chem. Int. Ed.* **2011**, *50*, 11210-11214; b) W.-M. Liu, S. P. Skinner, M. Timmer, A. Blok, M. A. S. Hass, D. V. Filippov, M. Overhand, M. Ubbink, *Chem. Eur. J.* **2014**, *20*, 6256-6258; c) M. L. Ludwiczek, M. Heller, T. Kantner, L. P. McIntosh, *J. Mol. Biol.* **2007**, *373*, 337-354.

[4] J. D. Swarbrick, P. Ung, M. L. Dennis, M. D. Lee, S. Chhabra, B. Graham, *Chem. Eur. J.* **2015**, *22*, 1228-1232.

[5] W. F. Vranken, W. Boucher, T. J. Stevens, R. H. Fogh, A. Pajon, M. Llinas, E. L. Ulrich, J. L. Markley, J. Ionides, E. D. Laue, *Proteins: Struct., Funct., Bioinf.* **2005**, *59*, 687-696.

[6] a) L. P. McIntosh, A. J. Wand, D. F. Lowry, A. G. Redfield, F. W. Dahlquist, *Biochemistry* **1990**, *29*, 6341-6362; b) L. A. Plesniak, L. P. McIntosh, W. W. Wakarchuk, *Protein Sci.* **1996**, *5*, 1118-1135.

[7] C. Schmitz, M. J. Stanton-Cook, X.-C. Su, G. Otting, T. Huber, *J. Biomol. NMR* **2008**, *41*, 179.

[8] L. H. Weaver, B. W. Matthews, *J. Mol. Biol.* **1987**, *193*, 189-199; b) G. Sidhu, S. G. Withers, N. T. Nguyen, L. P. McIntosh, L. Ziser, G. D. Brayer,

Biochemistry **1999**, *38*, 5346-5354; c) A. S. Maltsev, A. Grishaev, J. Roche, M. Zasloff, A. Bax, *J. Am. Chem. Soc.* **2014**, *136*, 3752-3755.

[9] Q. Bashir, A. N. Volkov, G. M. Ullmann, M. Ubbink, *J. Am. Chem. Soc.* **2010**, *132*, 241-247.

[10] a) J. L. Battiste, G. Wagner, *Biochemistry* **2000**, *39*, 5355-5365; b) J. Schilder, F. Löhr, H. Schwalbe, M. Ubbink, *FEBS Lett.* **2014**, *588*, 1873-1878.

[11] J. García de la Torre, M. L. Huertas, B. Carrasco, *J. Magn. Reson.* **2000**, *147*, 138-146.

[12] L. Liu, M. L. Quillin, B. W. Matthews, *Proc. Natl. Acad. Sci.* **2008**, *105*, 14406.

[13] D. W. Heinz, W. A. Baase, B. W. Matthews, *Proc. Natl. Acad. Sci.* **1992**, *89*, 3751-3755.

[14] M. L. Ludwiczek, I. D'Angelo, G. N. Yalloway, J. A. Brockerman, M. Okon, J. E. Nielsen, N. C. J. Strynadka, S. G. Withers, L. P. McIntosh, *Biochemistry* **2013**, *52*, 3138-3156.

[15] K. T. Dayie, G. Wagner, *J. Am. Chem. Soc.* **1997**, *119*, 7797-7806.

[16] K. Y. Huang, G. A. Amodeo, L. Tong, A. McDermott, *Protein Sci.* **2011**, *20*, 630-639.

Appendixes

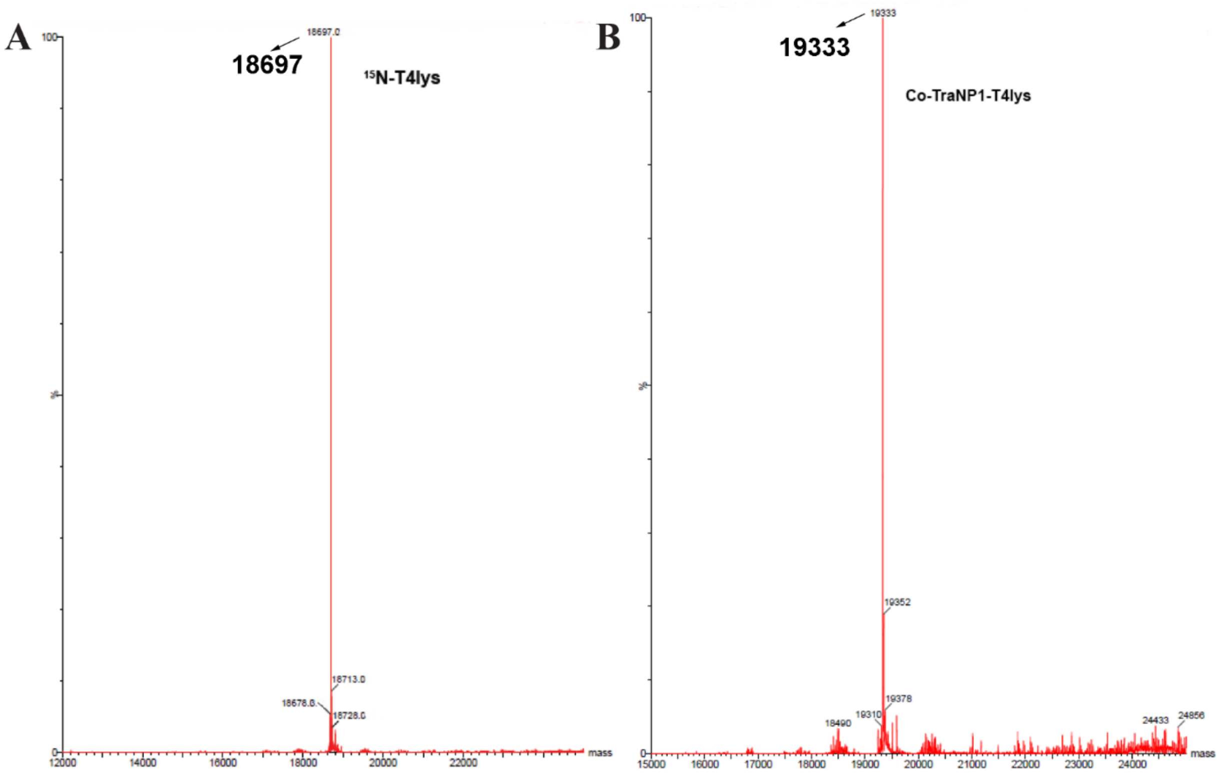

Appendix S1. ESI-TOF mass spectra of free ^{15}N-enriched K147C/T151C T4Lys mutant (A), and linked to Co-TraNP1.

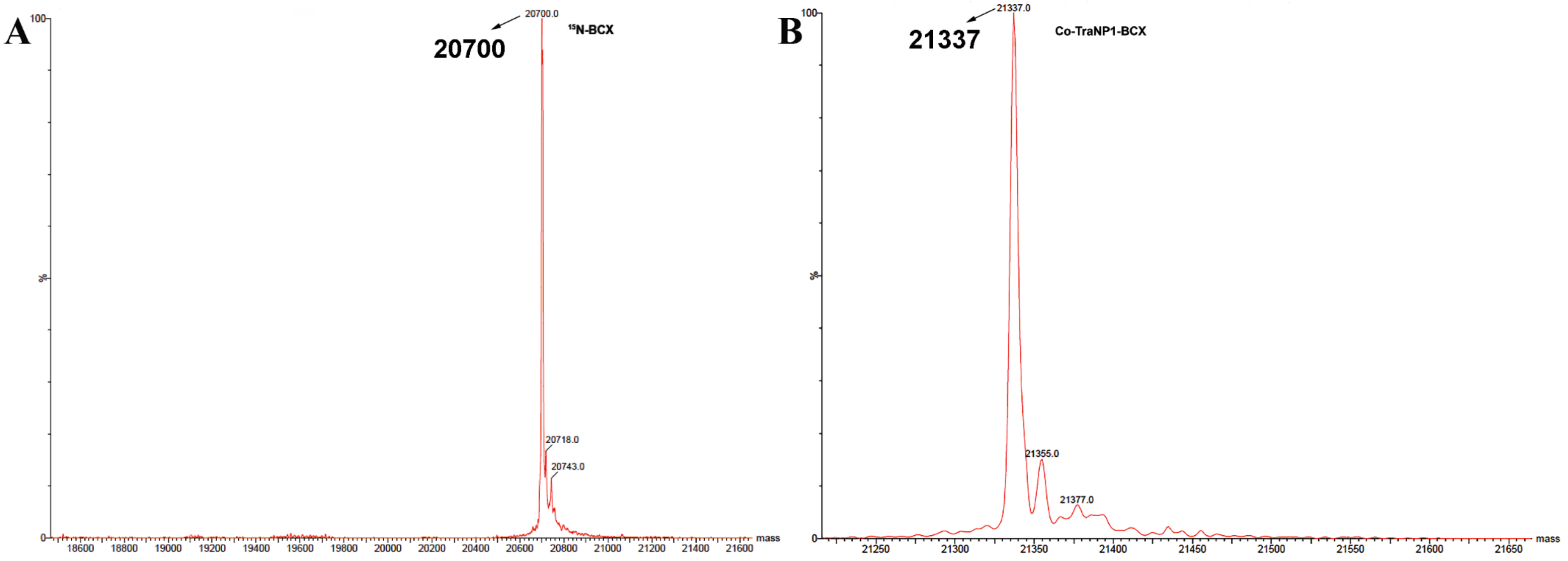

Appendix S2. ESI-TOF mass spectra of ^{15}N-enriched free T109C/T111C BCX mutant (A) and linked to Co(II)-TraNP1 (B).

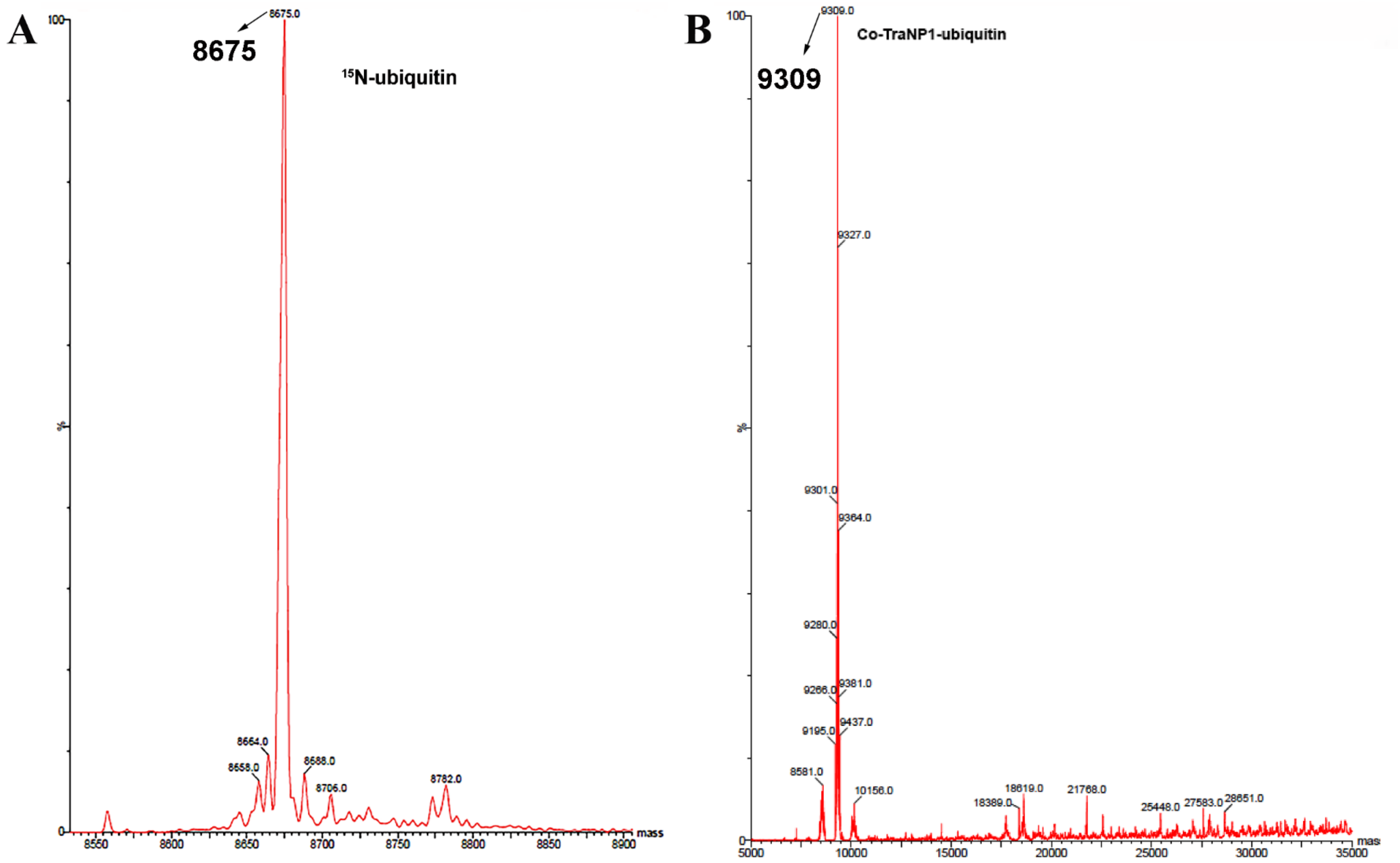

Appendix S3. ESI-TOF mass spectra of ^{15}N-enriched E24C/A28C ubiquitin mutant (A), and linked to Co-TraNP1 (B).

Appendixes S4 $^1H^N$ PCS of Co-TraNP1-SS linked to BCX.

Residue No.	Residue	PCS (ppm)	Residue	Residue	PCS (ppm)
2	SER	-0.014	68	LEU	-0.033
3	THR	-0.017	69	PHE	-0.058
5	TYR	-0.03	71	TRP	-0.048
6	TRP	-0.03	72	THR	-0.192
7	GLN	-0.035	73	ARG	-0.135
8	ASN	-0.032	74	SER	-0.132
9	TRP	-0.02	77	ILE	-0.3
10	THR	-0.03	81	VAL	-0.026
11	ASP	-0.031	82	VAL	-0.024
12	GLY	-0.021	83	ASP	-0.031
15	ILE	-0.017	85	TRP	-0.018
16	VAL	-0.04	89	ARG	-0.027
17	ASN	-0.017	94	TYR	0.36
19	VAL	-0.009	95	LYS	0.315
23	GLY	-0.012	97	THR	0.134
25	ASN	-0.018	98	VAL	0.071
26	TYR	-0.014	99	LYS	0.047
27	SER	-0.018	100	SER	0.025
28	VAL	-0.016	101	ASP	0.01
29	ASN	-0.018	102	GLY	0.012
30	TRP	-0.021	105	TYR	0.041
33	THR	-0.027	106	ASP	0.048
34	GLY	-0.023	107	ILE	0.121
35	ASN	-0.029	108	TYR	0.068
37	VAL	-0.037	113	TYR	-0.13
38	VAL	-0.028	114	ASN	0.15
40	LYS	-0.037	122	ARG	0.039
41	GLY	-0.031	123	THR	0.043
43	THR	-0.027	124	THR	0.157
46	SER	-0.048	130	SER	-0.032
48	PHE	-0.037	131	VAL	0.028
49	ARG	-0.032	133	GLN	0.057
50	THR	-0.016	146	PHE	-0.018
51	ILE	-0.017	150	VAL	-0.014
52	ASN	-0.014	152	ALA	0.038
53	TYR	-0.003	153	TRP	0.046
58	TRP	0.007	155	SER	0.097
59	ALA	-0.018	156	HIS	0.2
61	ASN	-0.013	157	GLY	0.251
62	GLY	-0.021	159	ASN	-0.084
63	ASN	-0.026	160	LEU	-0.091
64	GLY	-0.033	161	GLY	-0.212
65	TYR	-0.057	162	SER	-0.145
66	LEU	-0.037	163	ASN	-0.096
67	THR	-0.035	165	ALA	-0.097
167	GLN	-0.048	177	SER	-0.027

170	ALA	-0.038	179	SER	-0.019
174	TYR	-0.046	181	ASN	-0.016
175	GLN	-0.035	184	VAL	-0.018
176	SER	-0.029	185	TRP	-0.015

Appendixes S5 $^{1}H^{N}$ PCS of Co-TraNP1-RR linked to BCX

Residue No.	Residue type	PCS (ppm)	Residue No.	Residue type	PCS (ppm)
2	ASN	-0.168	71	VAL	-0.052
3	ILE	-0.13	72	ASP	-0.047
4	PHE	-0.115	73	ALA	-0.042
7	LEU	-0.171	74	ALA	-0.049
8	ARG	-0.126	75	VAL	-0.054
9	ILE	-0.158	76	ARG	-0.041
10	ASP	-0.136	77	GLY	-0.039
11	GLU	-0.098	78	ILE	-0.047
12	GLY	-0.049	79	LEU	-0.037
14	ARG	-0.013	80	ARG	-0.028
19	LYS	0.019	82	ALA	-0.021
20	ASP	0.011	83	LYS	-0.024
21	THR	0.028	84	LEU	-0.033
23	GLY	0.018	87	VAL	-0.022
24	TYR	0.018	88	TYR	-0.035
27	ILE	-0.011	89	ASP	-0.025
29	ILE	-0.033	91	LEU	-0.018
30	GLY	-0.022	93	ALA	-0.035
31	HIS	-0.022	96	ARG	-0.086
32	LEU	-0.022	99	LEU	-0.164
44	SER	-0.004	100	ILE	-0.165
46	LEU	-0.007	102	MET	-0.227
48	LYS	-0.008	104	PHE	-0.098
49	ALA	-0.011	105	GLN	-0.09
50	ILE	-0.011	106	MET	-0.082
51	GLY	-0.01	107	GLY	-0.058
52	ARG	-0.009	108	GLU	-0.049
53	ASN	-0.005	109	THR	-0.04
55	ASN	-0.003	110	GLY	-0.042
58	ILE	-0.007	112	ALA	-0.055
59	THR	-0.012	113	GLY	-0.055
60	LYS	-0.021	114	PHE	-0.06
61	ASP	-0.028	115	THR	-0.055
62	GLU	-0.021	116	ASN	-0.049
63	ALA	-0.025	117	SER	-0.025
64	GLU	-0.036	118	LEU	-0.051
65	LYS	-0.034	121	LEU	0.003
67	PHE	-0.035	124	LYS	0.063
68	ASN	-0.045	125	ARG	0.11
70	ASP	-0.04	126	TRP	0.25

129	ALA	0.137	165	ALA	-0.11
130	ALA	0.128	166	TYR	-0.082
133	LEU	-0.19	167	GLN	-0.067
135	LYS	-0.315	168	VAL	-0.051
137	ARG	-0.098	170	ALA	-0.051
139	TYR	-0.16	173	GLY	-0.069
149	VAL	-0.882	174	TYR	-0.063
155	THR	0.132	175	GLN	-0.058
157	THR	-0.133	176	SER	-0.043
160	LEU	-0.163	181	ASN	-0.02
161	GLY	-0.266	184	VAL	-0.023
162	SER	-0.18	185	TRP	-0.022
163	ASN	-0.125			

Appendixes S6. ^{1}HN PCS of Co-TraNP1-SS linked to T4Lys.

Residue No.	Residue type	PCS (ppm)	Residue No.	Residue	PCS (ppm)
2	ASN	-0.168	71	VAL	-0.052
3	ILE	-0.13	72	ASP	-0.047
4	PHE	-0.115	73	ALA	-0.042
7	LEU	-0.171	74	ALA	-0.049
8	ARG	-0.126	75	VAL	-0.054
9	ILE	-0.158	76	ARG	-0.041
10	ASP	-0.136	77	GLY	-0.039
11	GLU	-0.098	78	ILE	-0.047
12	GLY	-0.049	79	LEU	-0.037
14	ARG	-0.013	80	ARG	-0.028
19	LYS	0.019	82	ALA	-0.021
20	ASP	0.011	83	LYS	-0.024
21	THR	0.028	84	LEU	-0.033
23	GLY	0.018	87	VAL	-0.022
24	TYR	0.018	88	TYR	-0.035
27	ILE	-0.011	89	ASP	-0.025
29	ILE	-0.033	91	LEU	-0.018
30	GLY	-0.022	93	ALA	-0.035
31	HIS	-0.022	96	ARG	-0.086
32	LEU	-0.022	99	LEU	-0.164
44	SER	-0.004	100	ILE	-0.165
46	LEU	-0.007	102	MET	-0.227
48	LYS	-0.008	104	PHE	-0.098
49	ALA	-0.011	105	GLN	-0.09
50	ILE	-0.011	106	MET	-0.082
51	GLY	-0.01	107	GLY	-0.058
52	ARG	-0.009	108	GLU	-0.049
53	ASN	-0.005	109	THR	-0.04
55	ASN	-0.003	110	GLY	-0.042
58	ILE	-0.007	112	ALA	-0.055
59	THR	-0.012	113	GLY	-0.055
60	LYS	-0.021	114	PHE	-0.06
61	ASP	-0.028	115	THR	-0.055
62	GLU	-0.021	116	ASN	-0.049
63	ALA	-0.025	117	SER	-0.025
64	GLU	-0.036	118	LEU	-0.051
65	LYS	-0.034	121	LEU	0.003
67	PHE	-0.035	124	LYS	0.063
68	ASN	-0.045	125	ARG	0.11
70	ASP	-0.04	126	TRP	0.25

129	ALA	0.137	139	TYR	-0.16
130	ALA	0.128	149	VAL	-0.882
133	LEU	-0.19	155	THR	0.132
135	LYS	-0.315	157	THR	-0.133
137	ARG	-0.098			

Appendixes S7 $^{1}H^{N}$ PCS of Co-TraNP1-RR linked to T4Lys.

Residue No.	Residue type	PCS (ppm)	Residue No.	Residue type	PCS (ppm)
2	ASN	-0.178	77	GLY	-0.037
3	ILE	-0.133	78	ILE	-0.05
4	PHE	-0.122	79	LEU	-0.037
7	LEU	-0.171	80	ARG	-0.025
8	ARG	-0.132	82	ALA	-0.019
9	ILE	-0.166	83	LYS	-0.021
10	ASP	-0.139	84	LEU	-0.027
11	GLU	-0.096	87	VAL	-0.019
12	GLY	-0.059	88	TYR	-0.031
14	ARG	-0.012	89	ASP	-0.016
19	LYS	0.019	93	ALA	-0.034
20	ASP	0.013	96	ARG	-0.08
23	GLY	0.021	99	LEU	-0.172
24	TYR	0.018	100	ILE	-0.169
27	ILE	-0.01	102	MET	-0.178
29	ILE	-0.037	104	PHE	-0.093
30	GLY	-0.028	105	GLN	-0.096
31	HIS	-0.022	106	MET	-0.08
32	LEU	-0.023	107	GLY	-0.062
44	SER	-0.002	108	GLU	-0.045
45	GLU	0.025	109	THR	-0.041
51	GLY	-0.009	110	GLY	-0.039
52	ARG	-0.009	113	GLY	-0.057
53	ASN	-0.005	114	PHE	-0.064
58	ILE	-0.008	115	THR	-0.051
59	THR	-0.014	116	ASN	-0.05
60	LYS	-0.021	117	SER	-0.058
61	ASP	-0.031	118	LEU	-0.045
62	GLU	-0.024	120	MET	0.015
63	ALA	-0.026	121	LEU	0.021
64	GLU	-0.038	126	TRP	0.309
65	LYS	-0.036	129	ALA	0.217
67	PHE	-0.035	130	ALA	0.263
68	ASN	-0.049	133	LEU	-0.177
70	ASP	-0.036	135	LYS	-0.402
71	VAL	-0.053	137	ARG	-0.122
72	ASP	-0.048	139	TYR	-0.18
74	ALA	-0.051	142	THR	0.093
75	VAL	-0.06	149	VAL	-0.914
76	ARG	-0.038	155	THR	0.186

157	THR	-0.153

Appendixes S8 $^{1}H^{N}$ PCS of Co-TraNP1-SS linked to ubiquitin.

Residue No.	Residue type	PCS (ppm)	Residue No.	Residue type	PCS (ppm)
3	ILE	-0.065	68	HIS	-0.047
4	PHE	-0.05	69	LEU	-0.047
5	VAL	-0.053	70	VAL	-0.051
6	LYS	-0.037	71	LEU	-0.034
7	THR	-0.037	72	ARG	-0.027
13	ILE	-0.041			
14	THR	-0.036			
15	LEU	-0.061			
16	GLU	-0.069			
17	VAL	-0.07			
18	GLU	-0.145			
20	SER	-0.084			
21	ASP	-0.129			
22	THR	-0.368			
23	ILE	-0.136			
24	CYS	-0.29			
26	VAL	-0.391			
27	LYS	-0.454			
30	ILE	-0.259			
31	GLN	-0.293			
35	GLY	-0.058			
36	ILE	-0.116			
39	ASP	-0.229			
40	GLN	-0.134			
41	GLN	-0.104			
43	LEU	-0.029			
44	ILE	-0.048			
47	GLY	-0.007			
50	LEU	-0.028			
56	LEU	-0.121			
57	SER	-0.057			
58	ASP	-0.034			
59	TYR	-0.037			
61	ILE	-0.027			
62	GLN	-0.037			
63	LYS	-0.033			
64	GLU	-0.037			
65	SER	-0.04			
66	THR	-0.035			
67	LEU	-0.057			

Appendixes S9 $^{1}H^{N}$ PCS of Co-*tag 1* linked to ubiquitin.

Residue No.	Residue type	PCS (ppm)	Residue No.	Residue type	PCS (ppm)
2	GLN	-0.149	62	GLN	-0.203
3	ILE	-0.316	63	LYS	-0.136
4	PHE	-0.358	64	GLU	-0.205
5	VAL	-0.394	65	SER	-0.22
6	LYS	-0.382	66	THR	-0.238
7	THR	-0.306	67	LEU	-0.382
10	GLY	-0.191	68	HIS	-0.444
11	LYS	-0.174	69	LEU	-0.38
12	THR	-0.209	71	LEU	-0.276
13	ILE	-0.341			
15	LEU	-0.407			
16	GLU	-0.348			
18	GLU	-0.167			
20	SER	-0.007			
21	ASP	-0.1			
23	ILE	-0.821			
26	VAL	-2.573			
30	ILE	-2.165			
31	GLN	-2.221			
34	GLU	-0.549			
35	GLY	-0.484			
36	ILE	-0.57			
39	ASP	0.828			
41	GLN	-0.844			
43	LEU	-0.824			
44	ILE	-0.486			
45	PHE	-0.34			
47	GLY	-0.172			
50	LEU	-0.475			
51	GLU	-0.324			
52	ASP	-0.436			
55	THR	-0.029			
56	LEU	-0.285			
57	SER	-0.152			
58	ASP	-0.052			
59	TYR	-0.168			
60	ASN	-0.106			
61	ILE	-0.123			

Appendixes S10 $^{1}H^{N}$ PCS of Co-*tag 1* linked to T4Lys.

Residue No.	Residue type	PCS (ppm)	Residue No.	Residue type	PCS (ppm)
3	ILE	-0.124	114	PHE	-0.249
4	PHE	-0.087	115	THR	-0.194
30	GLY	-0.043	117	SER	-0.277
31	HIS	-0.053	118	LEU	-0.289
32	LEU	-0.078	120	MET	-0.228
33	LEU	-0.042	121	LEU	-0.316
67	PHE	-0.04	123	GLN	-0.171
70	ASP	-0.073	124	LYS	-0.185
71	VAL	-0.097	125	ARG	-0.185
72	ASP	-0.09	126	TRP	-0.167
73	ALA	-0.095	127	ASP	-0.105
74	ALA	-0.128			
75	VAL	-0.142			
76	ARG	-0.114			
77	GLY	-0.124			
79	LEU	-0.132			
80	ARG	-0.113			
82	ALA	-0.103			
83	LYS	-0.126			
85	LYS	-0.144			
87	VAL	-0.175			
88	TYR	-0.204			
89	ASP	-0.154			
90	SER	-0.157			
91	LEU	-0.187			
93	ALA	-0.105			
96	ARG	-0.321			
100	ILE	-0.413			
103	VAL	-0.388			
104	PHE	-0.301			
105	GLN	-0.319			
106	MET	-0.292			
107	GLY	-0.219			
108	GLU	-0.164			
109	THR	-0.15			
110	GLY	-0.171			
112	ALA	-0.21			
113	GLY	-0.201			

Appendixes S11 XPLOR script for PRE calculation parameters

```
! Energy minimization of metal pseudoatom position using PRE restraints
! Using T4 Lysozym (2LZM.pdb)
! file: pre_run.inp
set mess=off end
 set echo=off end
!----------------------------------------------------------------------
!----------------VARIABLES-----------------------------------------------
!----------------------------------------------------------------------
 eval ($a1= 3.0)   ! lower bound CL3
 eval ($a2= 3.0)   ! upper bound CL3
 eval ($a3= 15.0) ! (target distance CL1)
 eval ($a4= 12.0) ! lower bound CL1
 eval ($a5= 3.0)   ! upper bound CL1
 eval ($a6= 29.0) ! (target distance CL2)
 eval ($a7= 3.0)   ! lower bound CL2
 eval ($a8= 100.0)            ! upper bound CL2
 eval ($a9= 0.05) ! general scaling
 eval ($a10= 1.0) ! scaling CL1
 eval ($a11= 1.0) ! scaling CL2
 eval ($a12= 1.0) ! scaling CL3
 eval ($a13= 100)! nr steps EM
 eval ($a14= 10)  ! nr runs EM
 eval ($tau_c= 8) ! tau-c in ns
 eval ($S01 = 5/2)            ! S-factor for Mn2+
 eval ($frl=1.0)    ! fraction of spin labelled TOAC peptide
 eval ($field=600.13)        ! field in MHz
 eval ($taufactor = 1e-9*(4*$tau_c+(3*$tau_c/(1+($field*1E-3*2*3.14*$tau_c)^2))))
 eval ($Sfactor = $S01*(1+$S01))

!----------------------------------------------------------------------
!----------------RESTRAINTS ---------------------------------------------
!----------------------------------------------------------------------

 !DEFINE NMR DERIVED DISTANCES FOR MA-NH
 @restraints.xpl

!----------------------------------------------------------------------
!----------------RUN ----------------------------------------------------
!----------------------------------------------------------------------

 constraints fix (not name MA) end
 constraints interactions (name MA) (name *) end
 flag exclude * include noe end
 set mess=off end
 set echo=off end
 eval ($b2 = 1)
 while ($b2 LE $a14) loop RUNS
 vector do (X= 300*RAND - 150) (name MA)
 vector do (Y= 300*RAND - 150) (name MA)
 vector do (Z= 300*RAND - 150) (name MA)
 !          ! write starting coordinates of MA to file
 !vector show elem (x) (name MA)
 !eval ($b4=$RESULT)
 !vector show elem (y) (name MA)
 !eval ($b5=$RESULT)
```

```
!vector show elem (z) (name MA)
!eval ($b6=$RESULT)
!set display=ma_coor end
!display $b2    $b4    $b5    $b6
!set display=OUTPUT end
minimize powell
            drop=10
            nprint=10
            nstep=$a13
            tolgradient=0.0001
end
eval ($b3="Mapos."+encode($b2))
write coord output=$b3 sele= (name MA) end

   !calculate distances and store

for $ide in ID (store1 or store3 ore store5)
  loop store_dist
      eval ($distave = 0)
      eval ($preave = 0)
      pick bond (name MA) (ID $ide) geom
      eval ($dist01 = $RESULT)
            vector show elem (store7) (ID $ide)
      eval ($distave = $RESULT+$dist01)
            vector do (store7=$distave) (ID $ide)
            eval ($precalc= 1.65*1E-44*$Sfactor*$taufactor*$frl/((1e-10*$dist01)^(6)))
      vector show elem (store8) (ID $ide)
      eval ($preave = $RESULT + $precalc)
      vector do (store8=$preave) (ID $ide)
  end loop store_dist
eval ($b2 = $b2 +1)
end loop RUNS
!write coord output=T4lys_metal_opt.pdb end
energy end
set display=output.txt end
display Average distances between HN and metal based on PRE data
display $DATE $TIME Energy: $NOE
display res          dist(PRE)          dist(Calc)          PRE(exp)          PRE(calc)
set display=OUTPUT end

for $ide in ID (store1 or store3 ore store5)
  loop recall_dist
       eval ($d01 = 0)
       eval ($d02 = 0)
       eval ($d03 = 0)
       eval ($d04 = 0)
       eval ($d05 = 0)
       vector show elem (resi) (ID $ide)
       eval ($d01 = $RESULT)
       vector show elem (store2) (ID $ide)
       if ($RESULT>0) then eval ($d02 = $RESULT) end if
       vector show elem (store4) (ID $ide)
       if ($RESULT>0) then eval ($d02 = $RESULT) end if
       vector show elem (store6) (ID $ide)
       if ($RESULT>0) then eval ($d02 = $RESULT) end if
       vector show elem (store7) (ID $ide)
       eval ($d03 = $RESULT/$a14)            !sum of distances / number of runs
```

```
        vector show elem (store1) (ID $ide)
        if ($RESULT>0) then eval ($d04 = $RESULT) end if
        vector show elem (store3) (ID $ide)
        if ($RESULT>0) then eval ($d04 = $RESULT) end if
        vector show elem (store5) (ID $ide)
        if ($RESULT>0) then eval ($d04 = $RESULT) end if
        vector show elem (store8) (ID $ide)
        eval ($d05 = $RESULT/$a14)          !sum of PREs / number of runs
        set display=output.txt end
        display $d01         $d02     $d03     $d04     $d05
        set display=OUTPUT end
    end loop recall_dist
stop
```

Appendix S12 chemical NMR data

1H NMR spectra of compound 4 (ss)

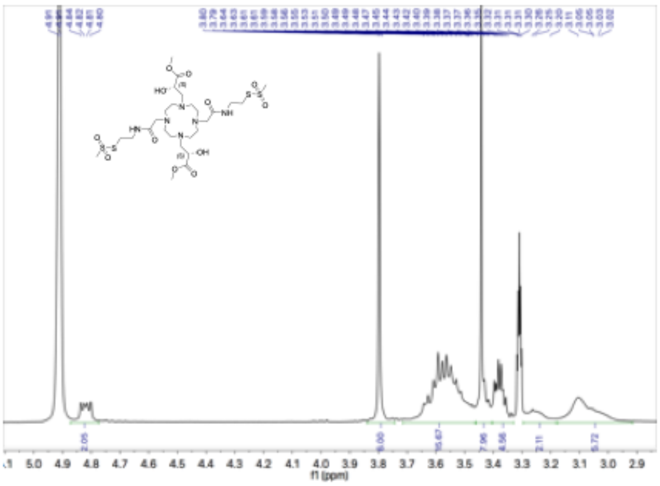

1H NMR spectra of compound 1

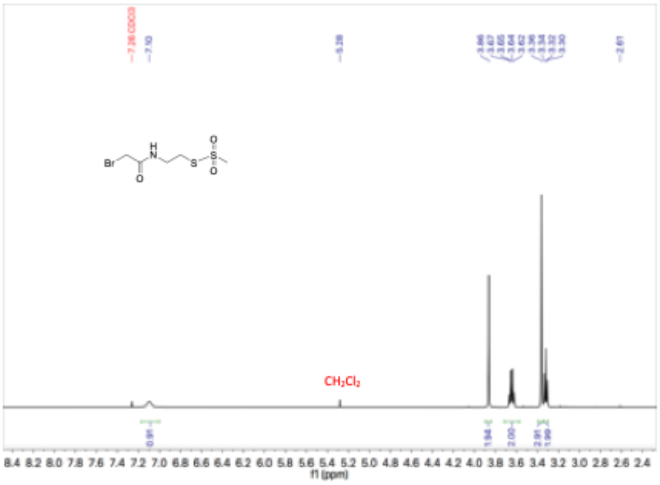

13C NMR spectra of compound 1

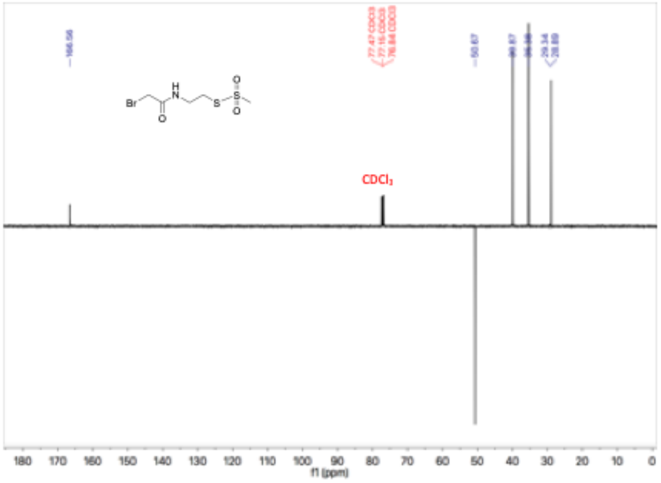

1H NMR spectra of compound 3 (ss)

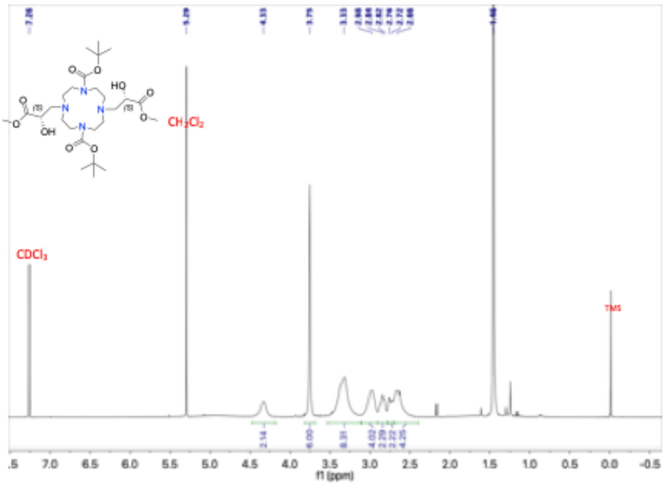

13C NMR spectra of compound 3 (ss)

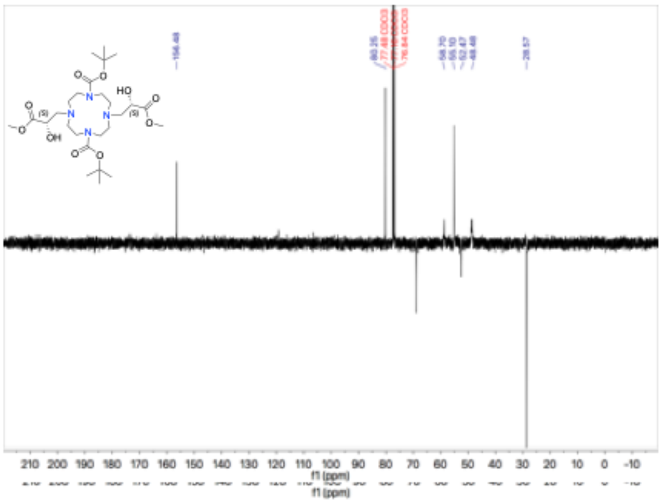

1H-1H COSY NMR spectra of compound 3 (ss)

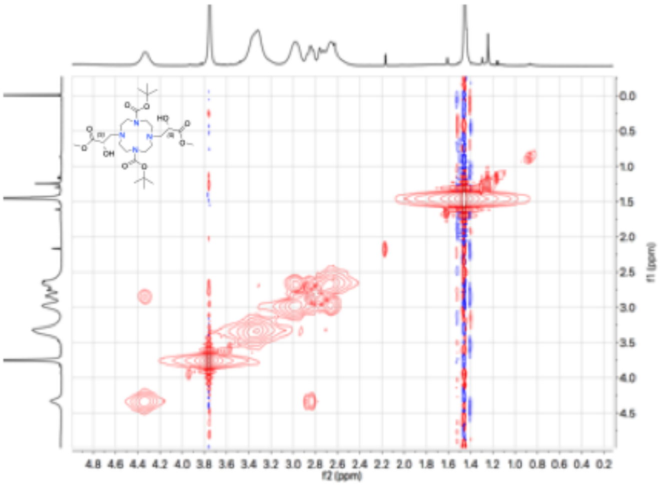

1H-13C HSQC NMR spectra of compound 3 (ss)

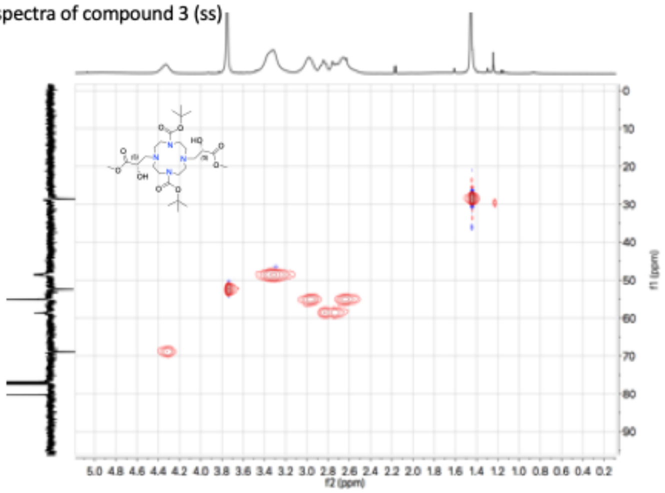

1H-13C HSQC NMR spectra of compound 4 (ss)

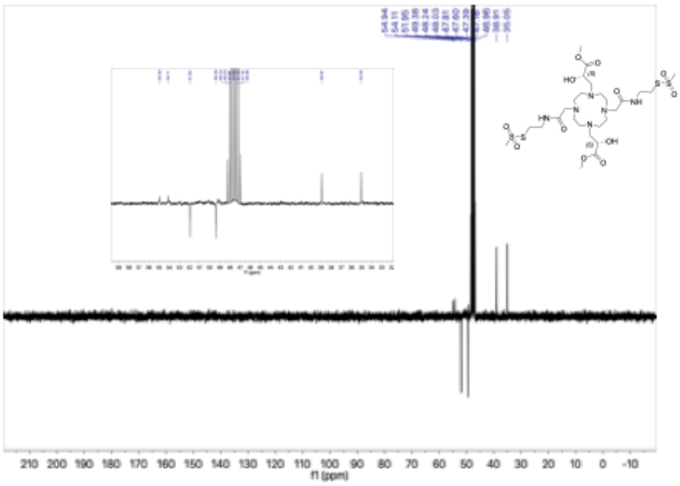

1H-1H COSY NMR spectra of compound 4 (ss)

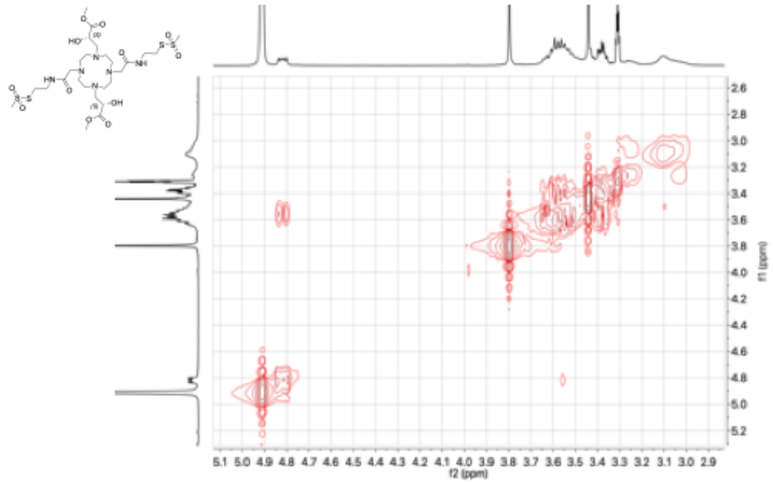

1H-13C HSQC NMR spectra of compound 4 (ss)

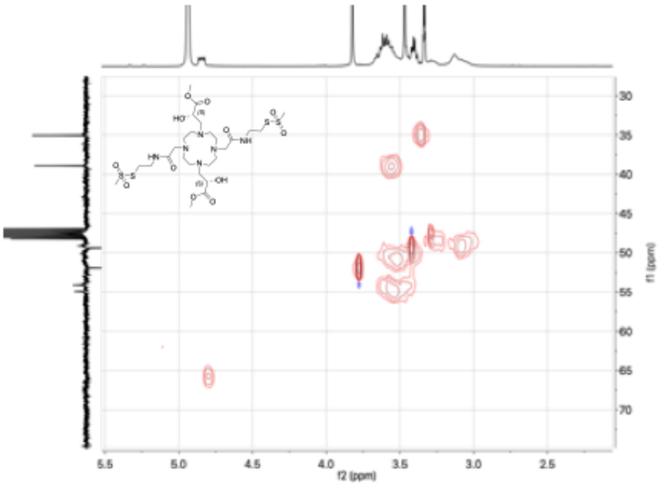

343K, 1H NMR spectra of TraNP1 (ss)

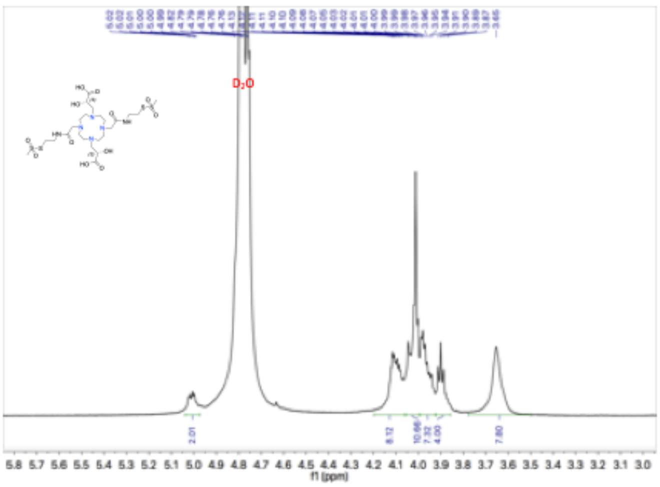

343K, 13C NMR spectra of TraNP1(ss)

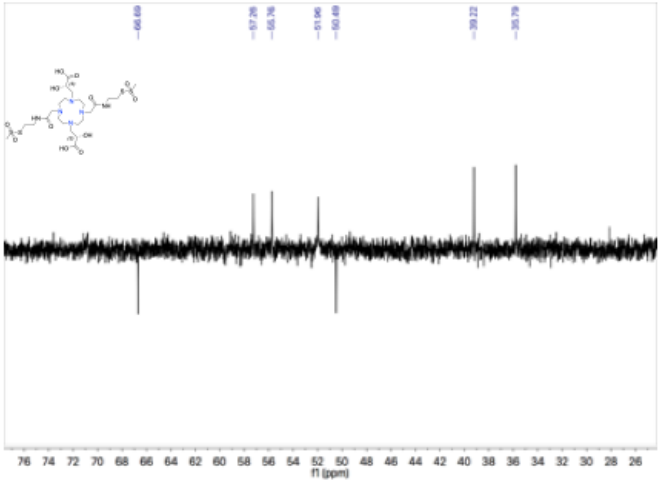

343K, 1H-1H COSY NMR spectra of TraNP1(ss)

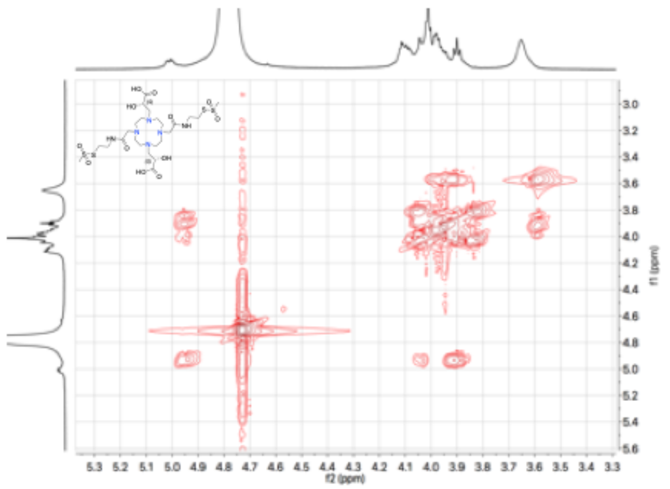

343K, 1H-13C HSQC NMR spectra of TraNP1 (ss)

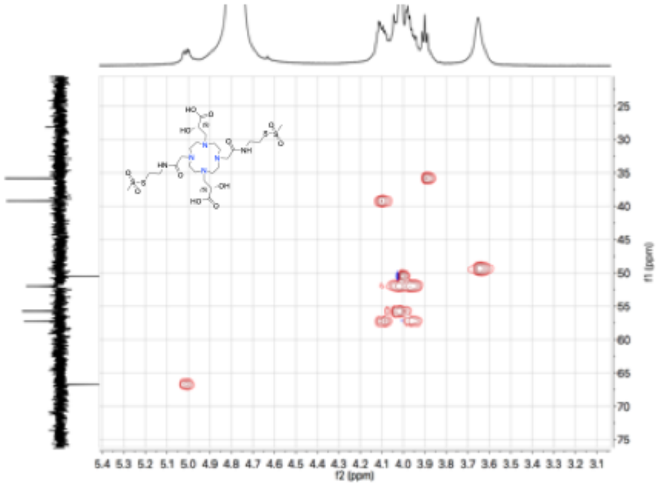

1H NMR spectra of compound 3 (rr)

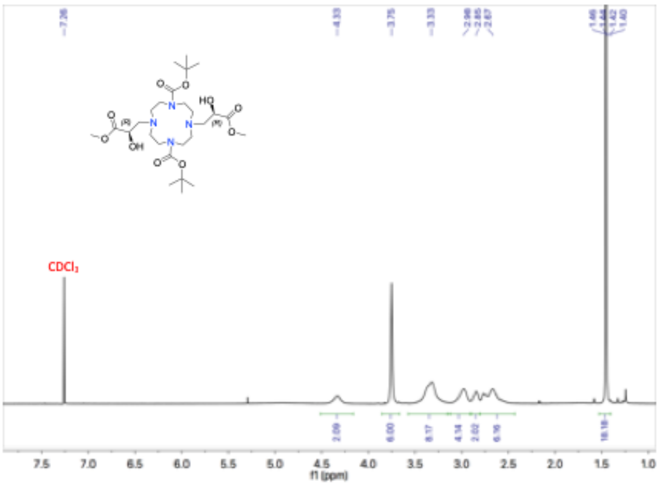

13C NMR spectra of compound 3 (rr)

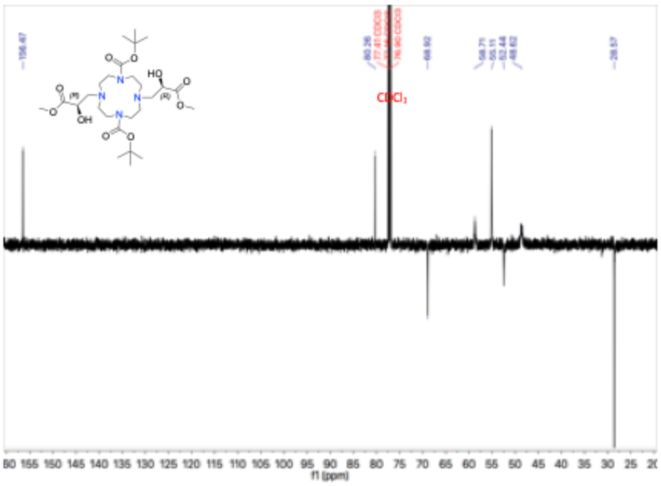

1H-1H COSY NMR spectra of compound 3 (rr)

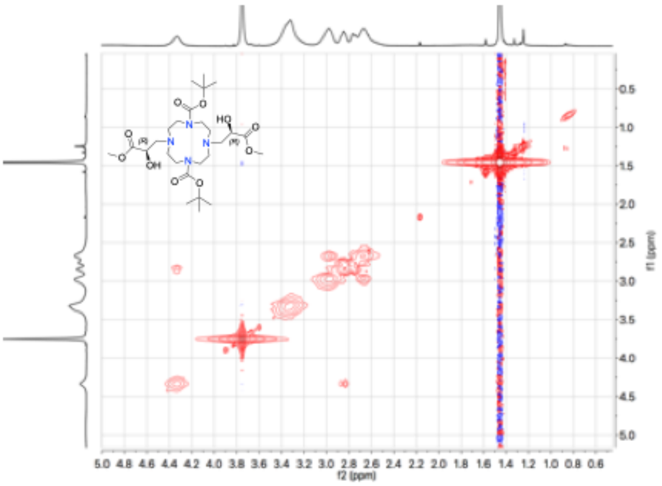

1H-13C HSQC NMR spectra of compound 3 (rr)

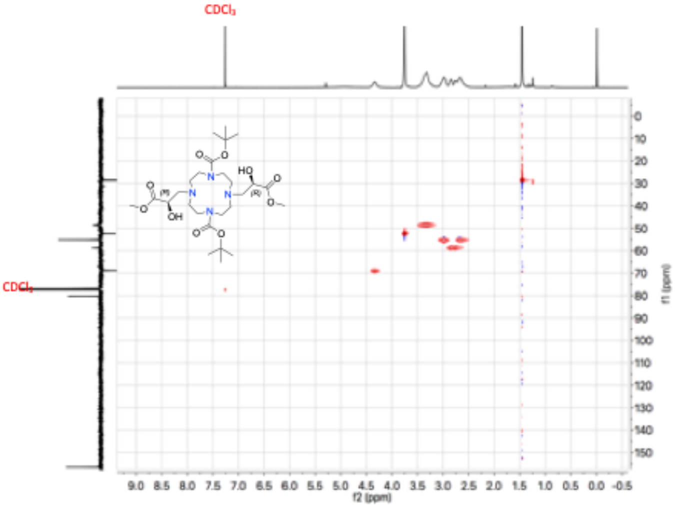

343K, 1H NMR spectra of compound 4 (rr)

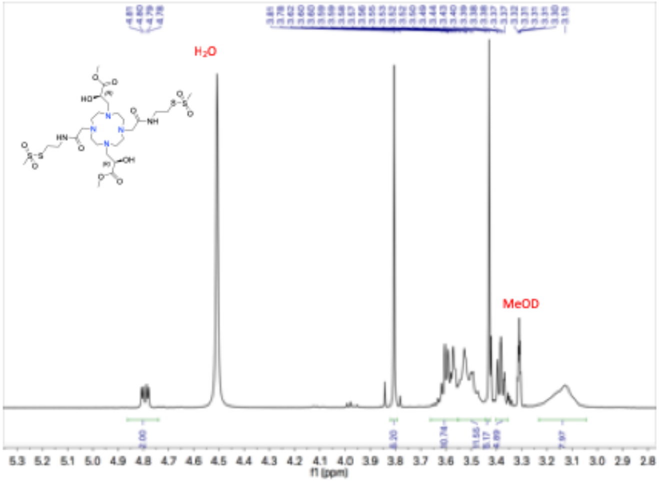

343K, 13C NMR spectra of compound 4 (rr)

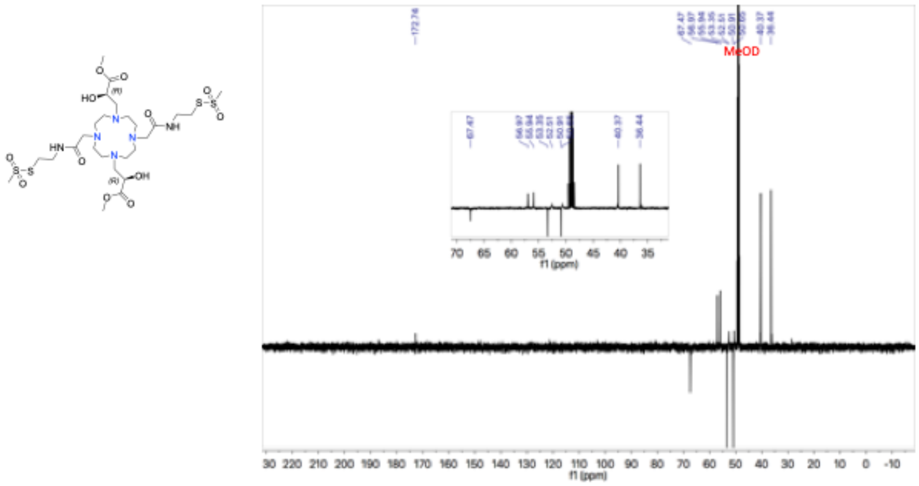

343K, 1H-1H COSY NMR spectra of compound 4 (rr)

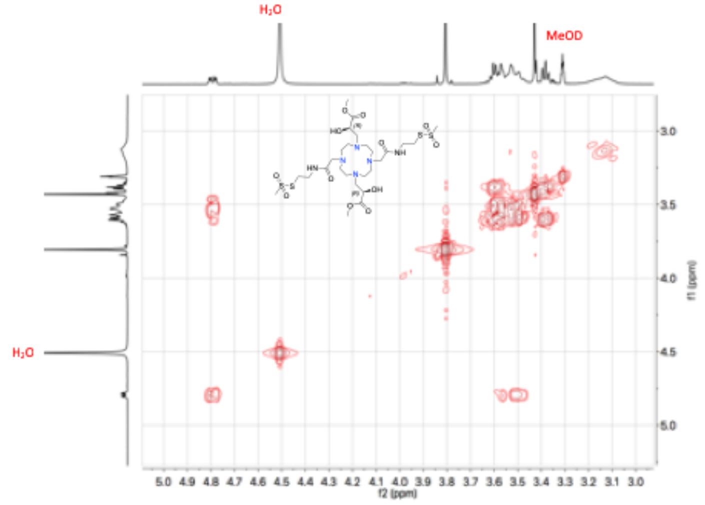

343K, 1H-13C HSQC NMR spectra of compound 4 (rr)

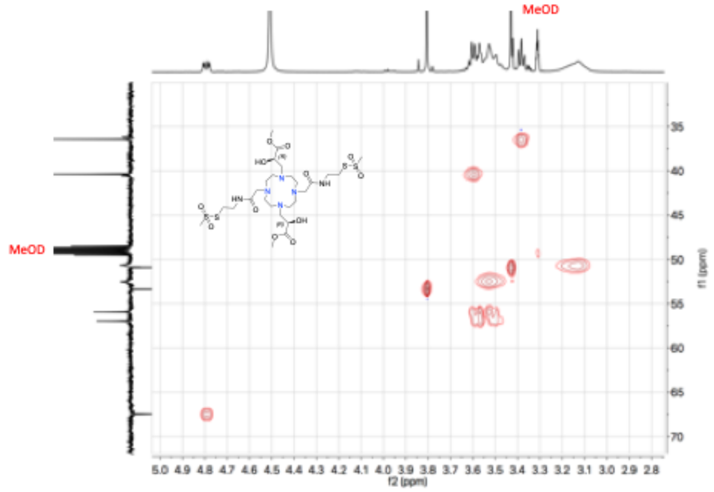

343K, 1H NMR spectra of TraNP1 (rr)

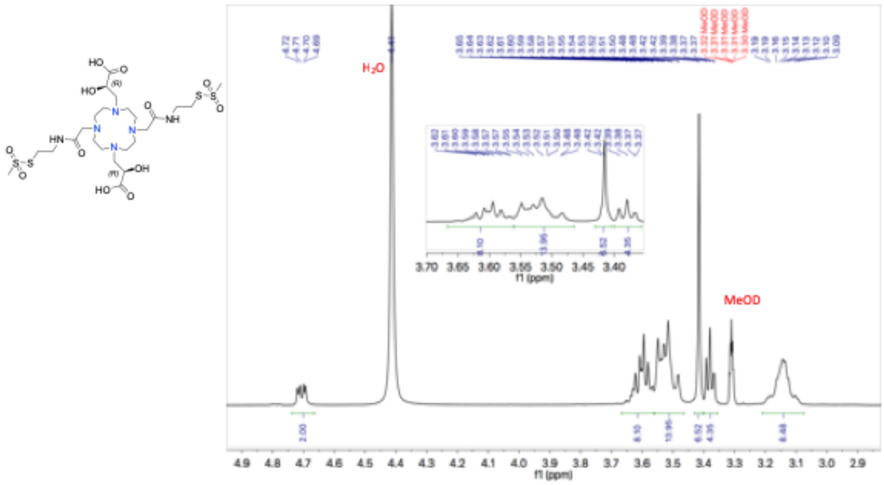

343K, 13C NMR spectra of TraNP1 (rr)

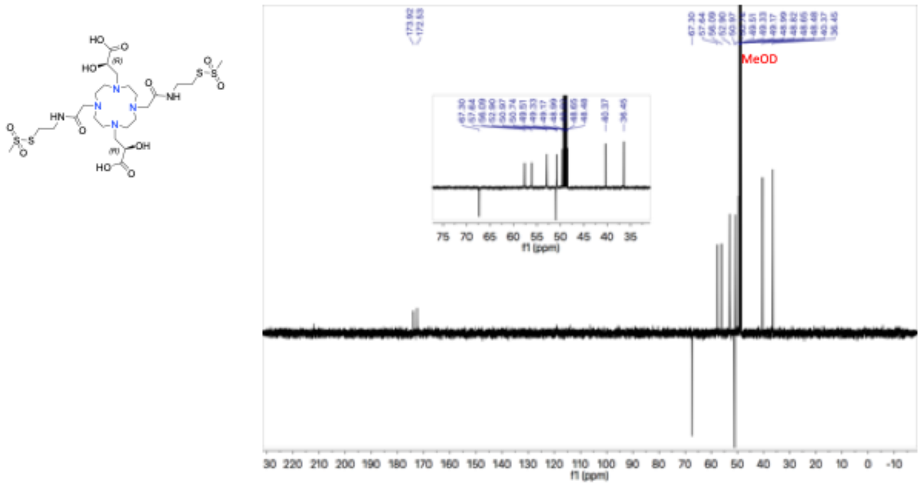

343K, 1H-1H NMR spectra of TraNP1 (rr)

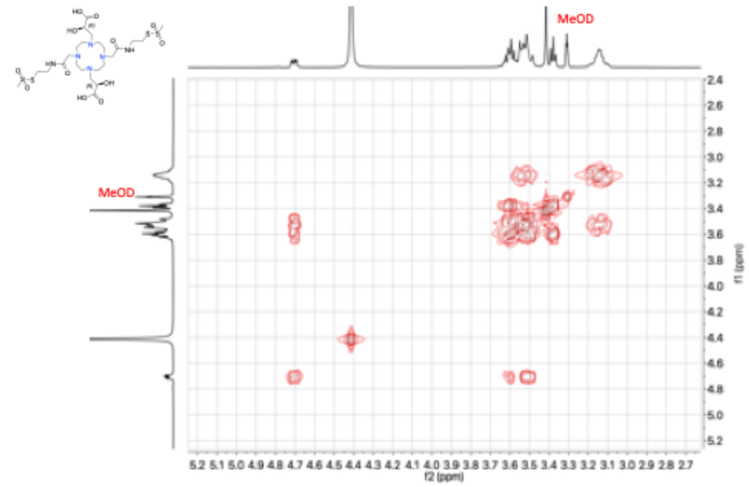

343K, 1H-13C NMR spectra of TraNP1 (rr)

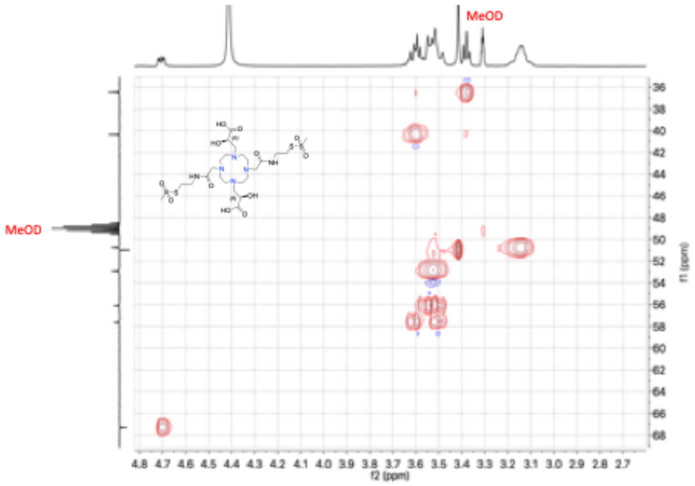

1H NMR spectra of compound 7

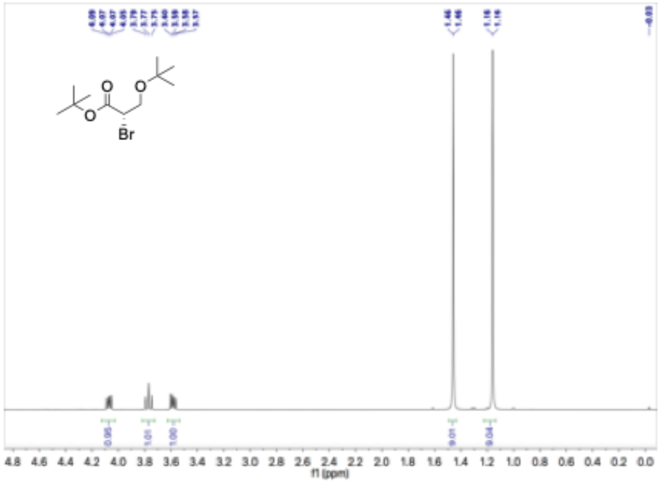

13C NMR spectra of compound 7

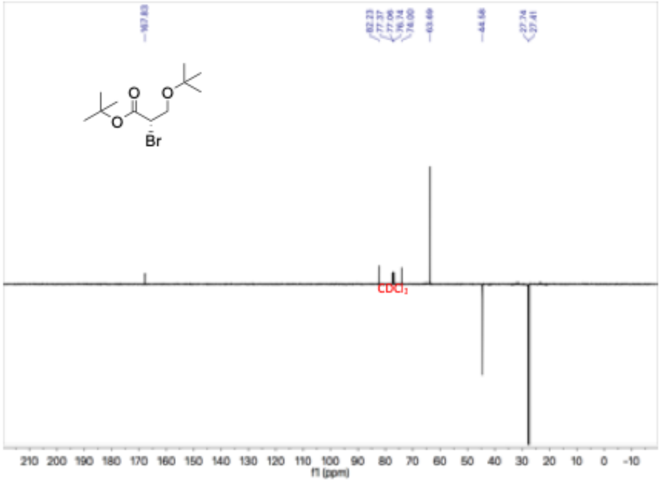

1H NMR spectra of TraNP3

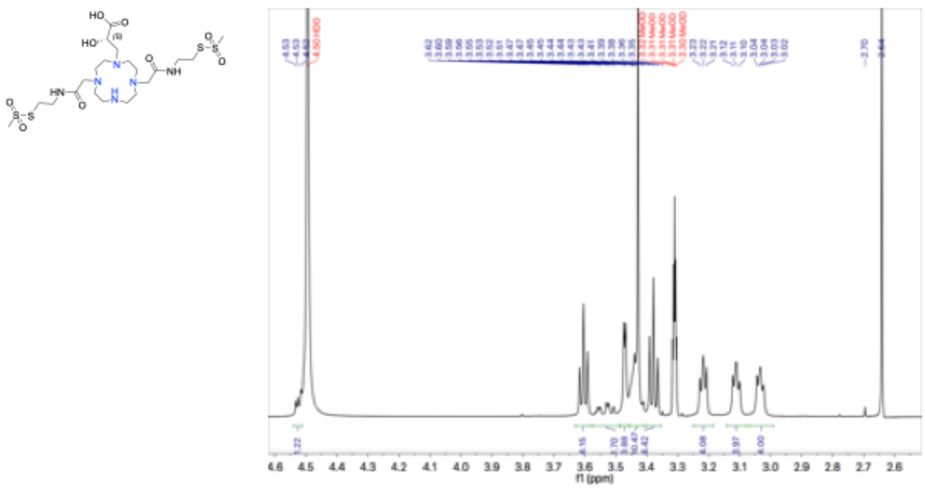

C NMR spectra of TraNP3

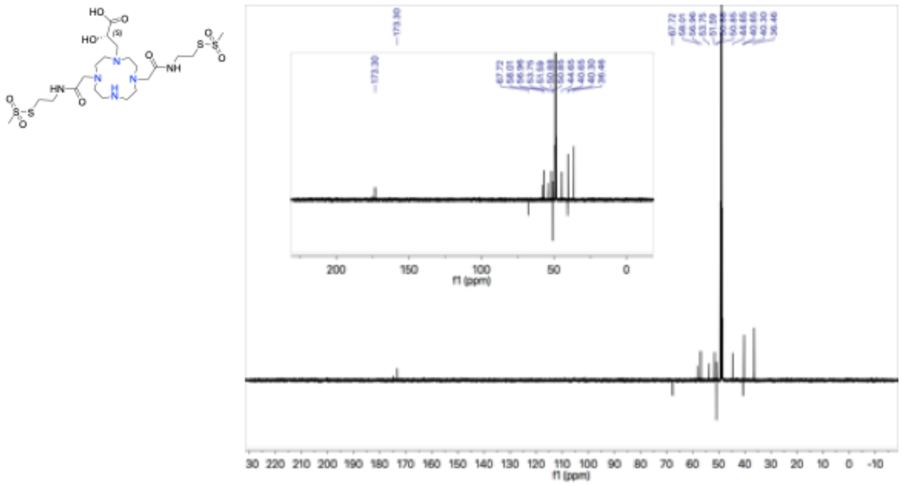

1H-1H COSY NMR spectra of TraNP3

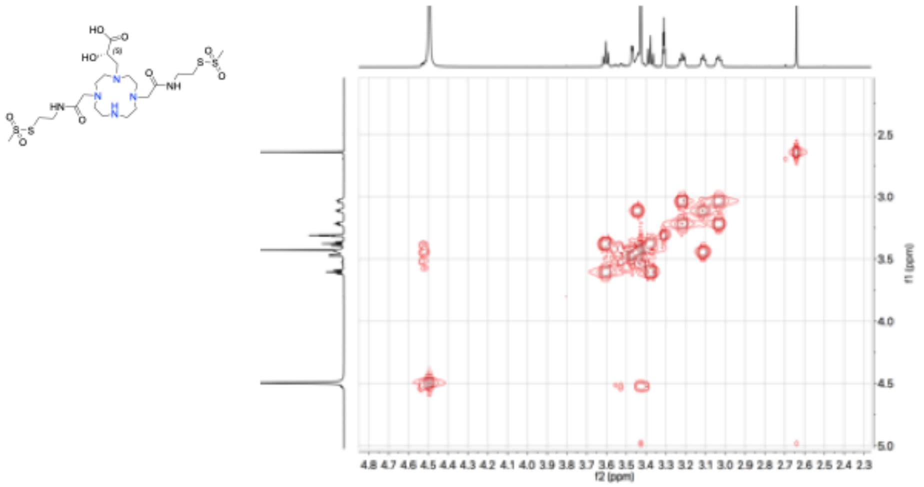

13C-1H HSQC NMR spectra of TraNP3

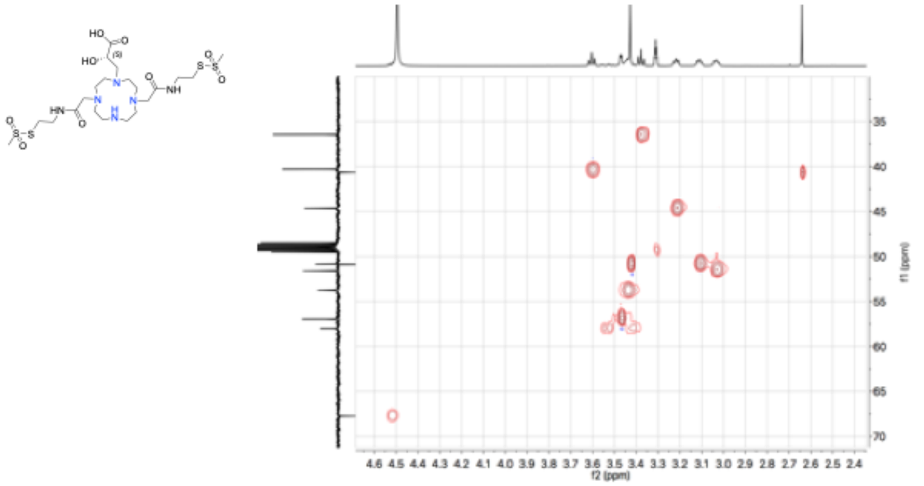

1H NMR spectra of TraNP2

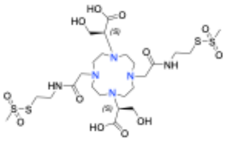

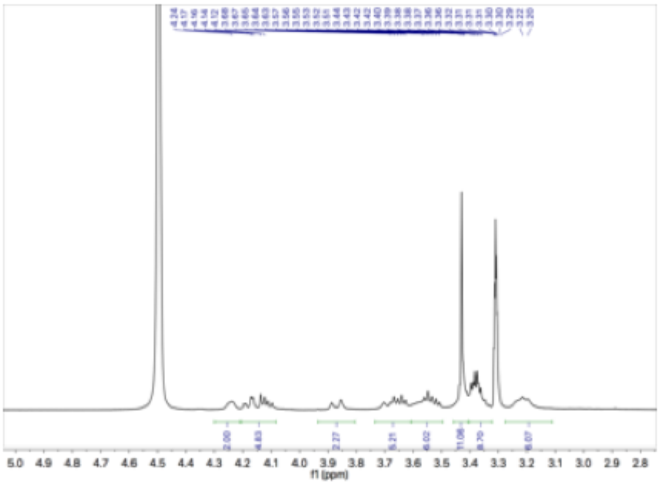

13C NMR spectra of TraNP2

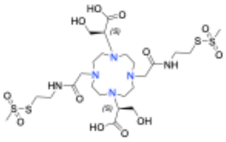

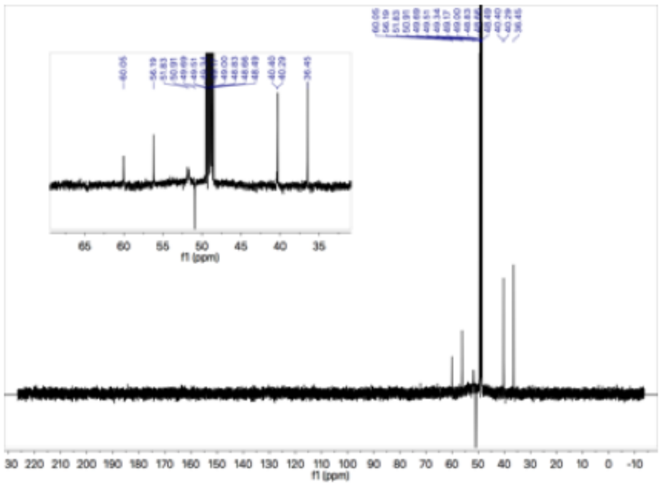

1H-1H COSY NMR spectra of TraNP2

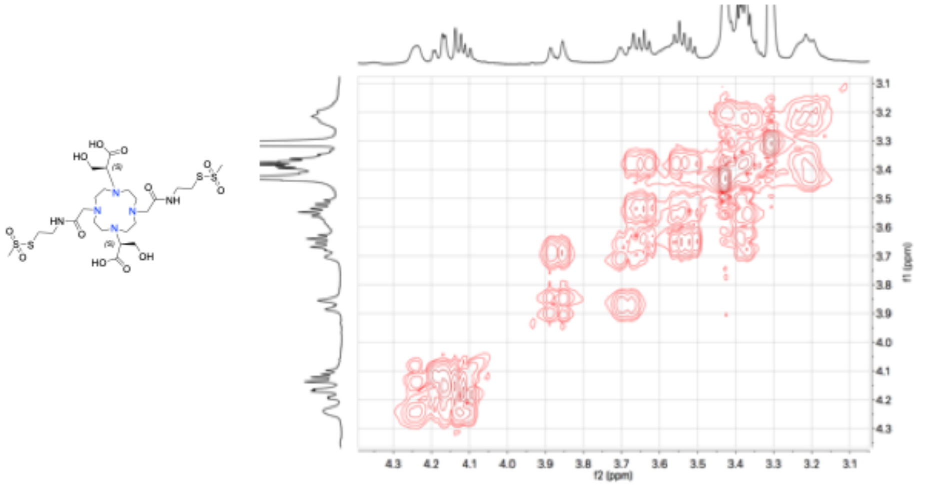

13C-1H HSQC NMR spectra of TraNP2

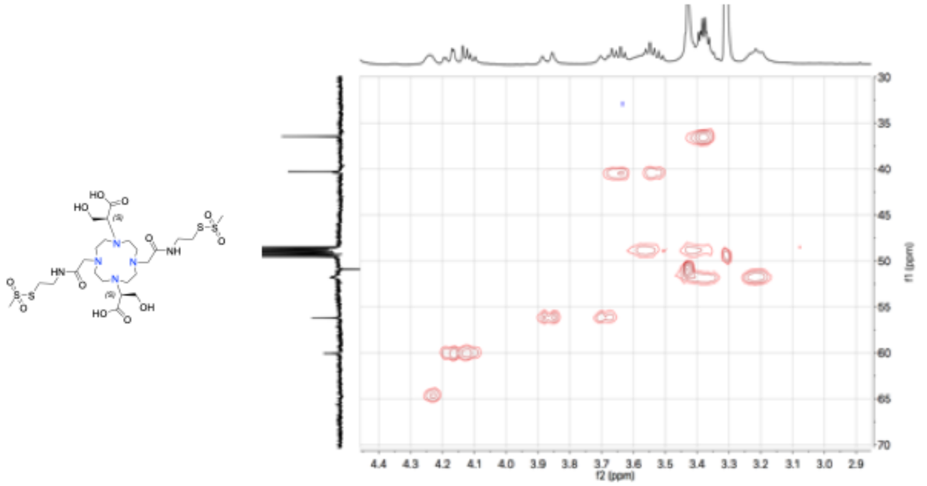

1H NMR spectra of compound 5

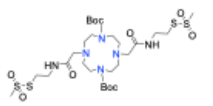

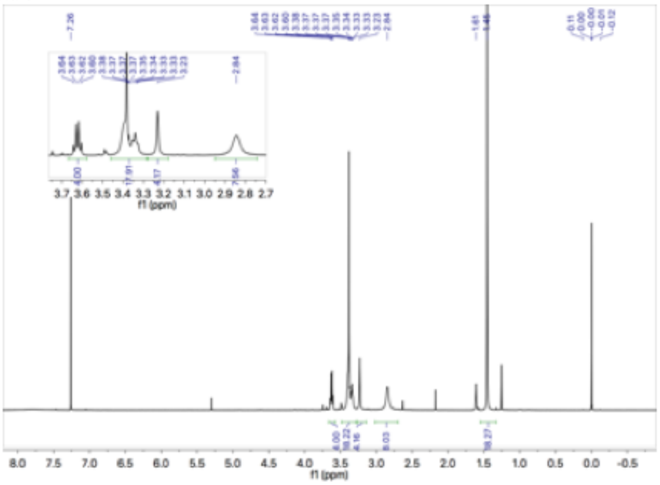

13C NMR spectra of compound 5

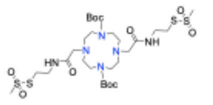

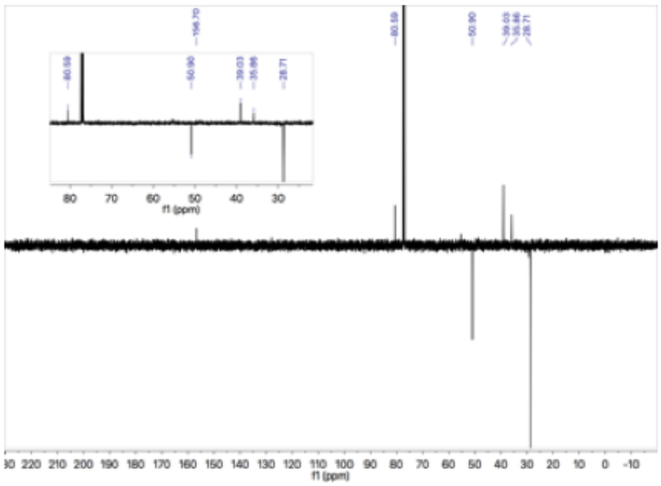

13C-1H NMR spectra of compound 5

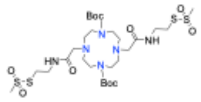

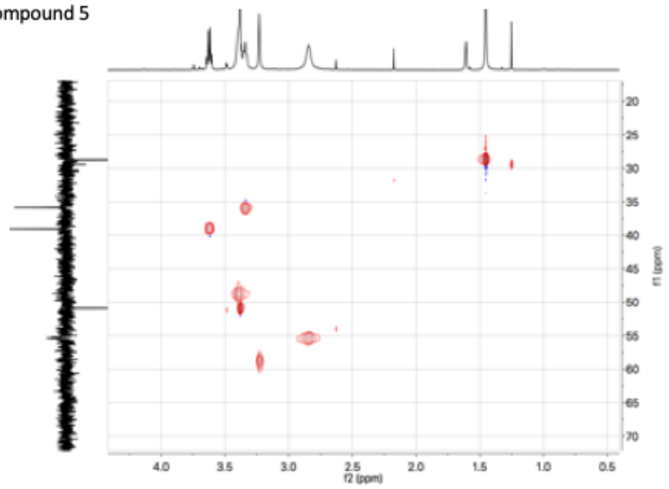

1H NMR spectra of TraNP4

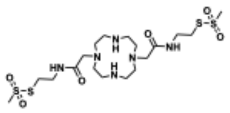

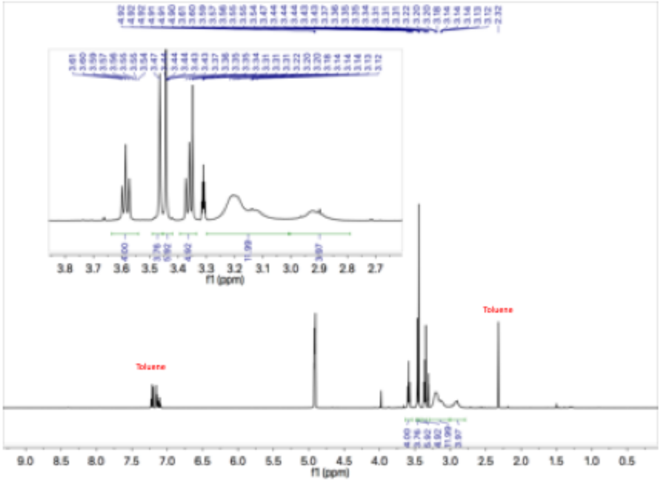

13C NMR spectra of TraNP4

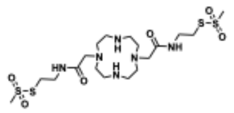

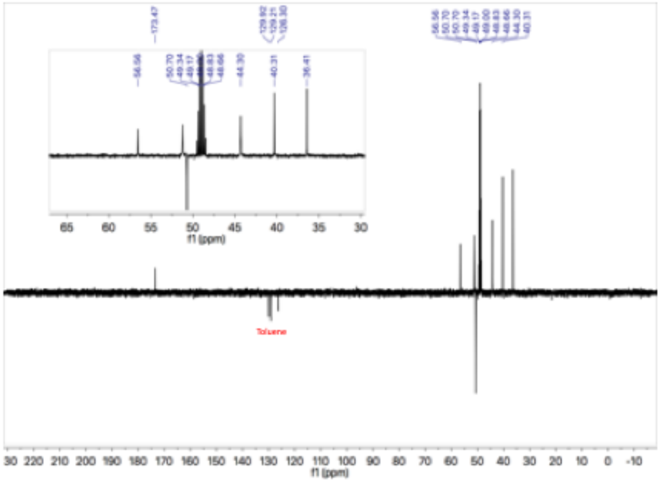

13C-1H NMR spectra of TraNP4

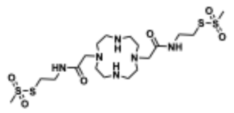

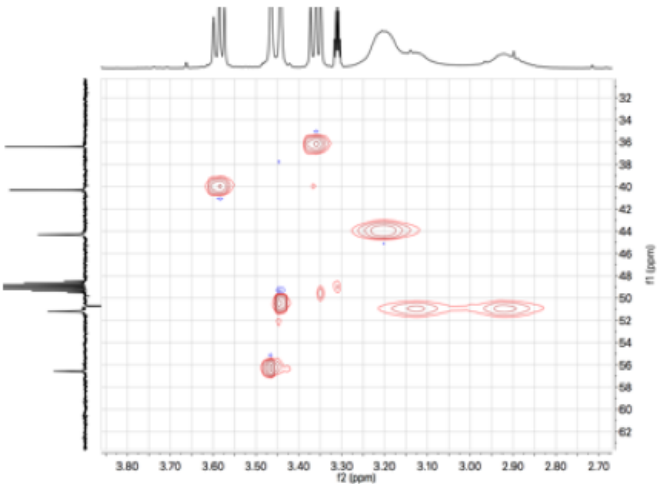

1H NMR spectra of compound 9

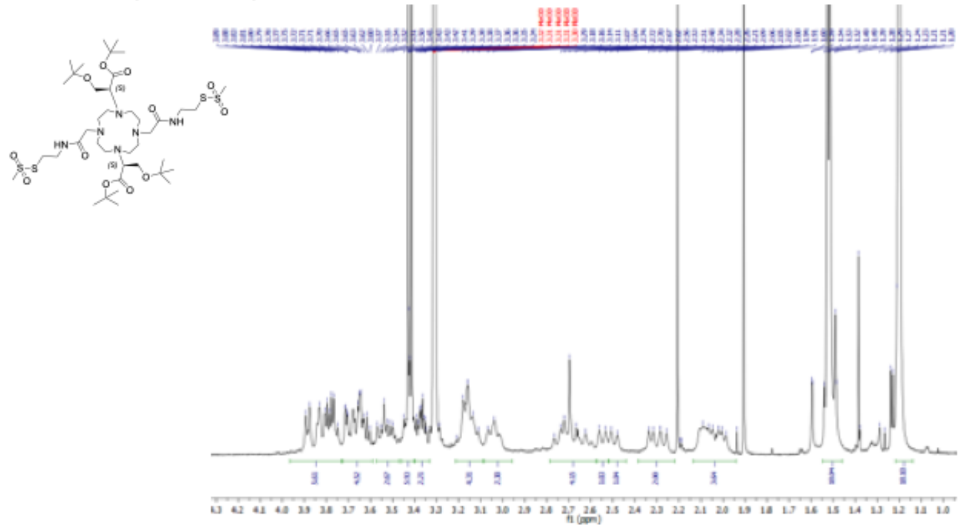

13C NMR spectra of compound 9

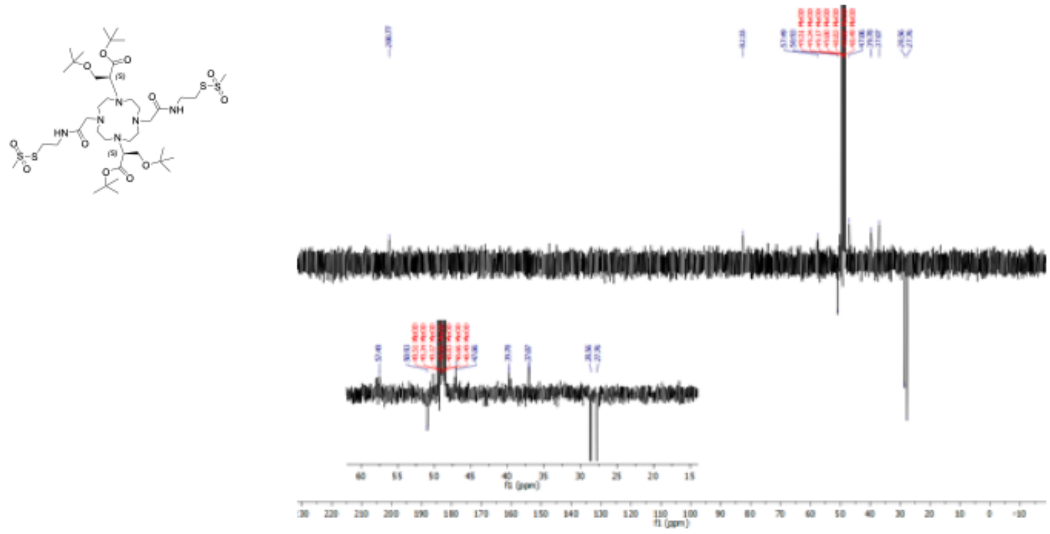

1H-1H cosy NMR spectra of compound 9

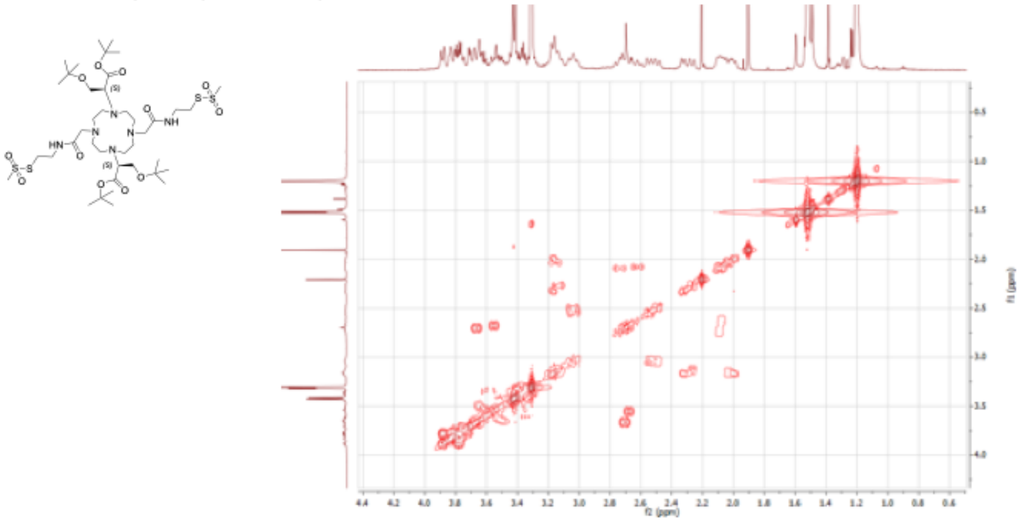

1H-13C HSQC NMR spectra of compound 9

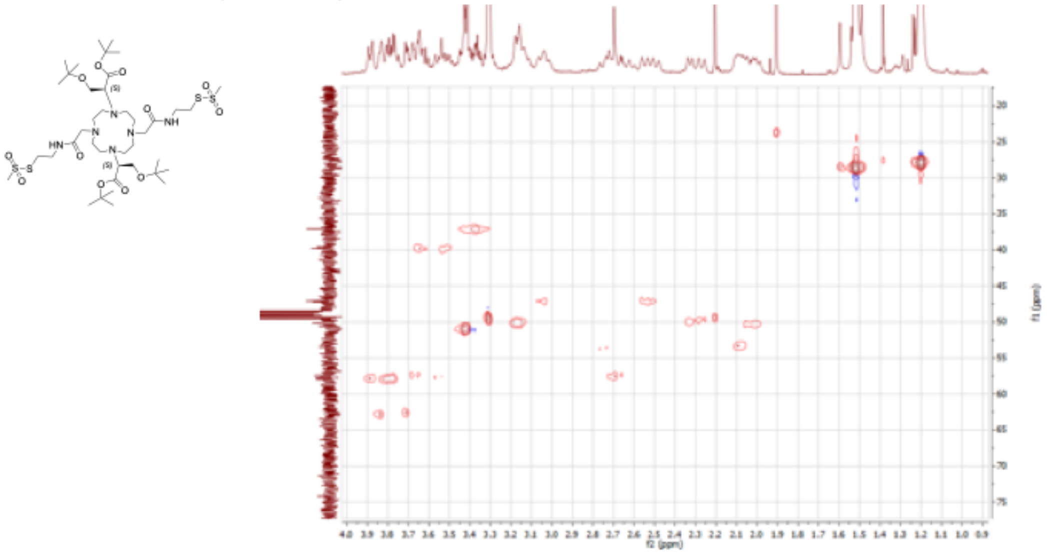

Supplement: Supplementary file 1 — Supplementary [file ANIE-58-13093-s001.pdf]
